# Metabolomics-directed nanotechnology in viral diseases management: COVID-19 a case study

**DOI:** 10.1007/s43440-023-00517-w

**Published:** 2023-08-16

**Authors:** Marwa O. El-Derany, Diana M. F. Hanna, John Youshia, Enas Elmowafy, Mohamed A. Farag, Samar S. Azab

**Affiliations:** 1 Department of Biochemistry, Faculty of Pharmacy, Ain Shams University, Cairo, Egypt; 2 Department of Pharmacology and Toxicology, Faculty of Pharmacy, Ain Shams University, 11566 Cairo, Egypt; 3 Department of Pharmaceutics and Industrial Pharmacy, Faculty of Pharmacy, Ain Shams University, Cairo, Egypt; 4 Pharmacognosy Department, College of Pharmacy, Cairo University, Kasr El-Aini St., P.B. 11562, Cairo, Egypt

**Keywords:** SARS-CoV-2, COVID-19, Nanoparticles, Metabolomics, Viral infections

## Abstract

The severe acute respiratory syndrome coronavirus 2 (SARS-CoV-2) is currently regarded as the twenty-first century’s plague accounting for coronavirus disease 2019 (COVID-19). Besides its reported symptoms affecting the respiratory tract, it was found to alter several metabolic pathways inside the body. Nanoparticles proved to combat viral infections including COVID-19 to demonstrate great success in developing vaccines based on mRNA technology. However, various types of nanoparticles can affect the host metabolome. Considering the increasing proportion of nano-based vaccines, this review compiles and analyses how COVID-19 and nanoparticles affect lipids, amino acids, and carbohydrates metabolism. A search was conducted on PubMed, ScienceDirect, Web of Science for available information on the interrelationship between metabolomics and immunity in the context of SARS-CoV-2 infection and the effect of nanoparticles on metabolite levels. It was clear that SARS-CoV-2 disrupted several pathways to ensure a sufficient supply of its building blocks to facilitate its replication. Such information can help in developing treatment strategies against viral infections and COVID-19 based on interventions that overcome these metabolic changes. Furthermore, it showed that even drug-free nanoparticles can exert an influence on biological systems as evidenced by metabolomics.

**Graphical abstract:**

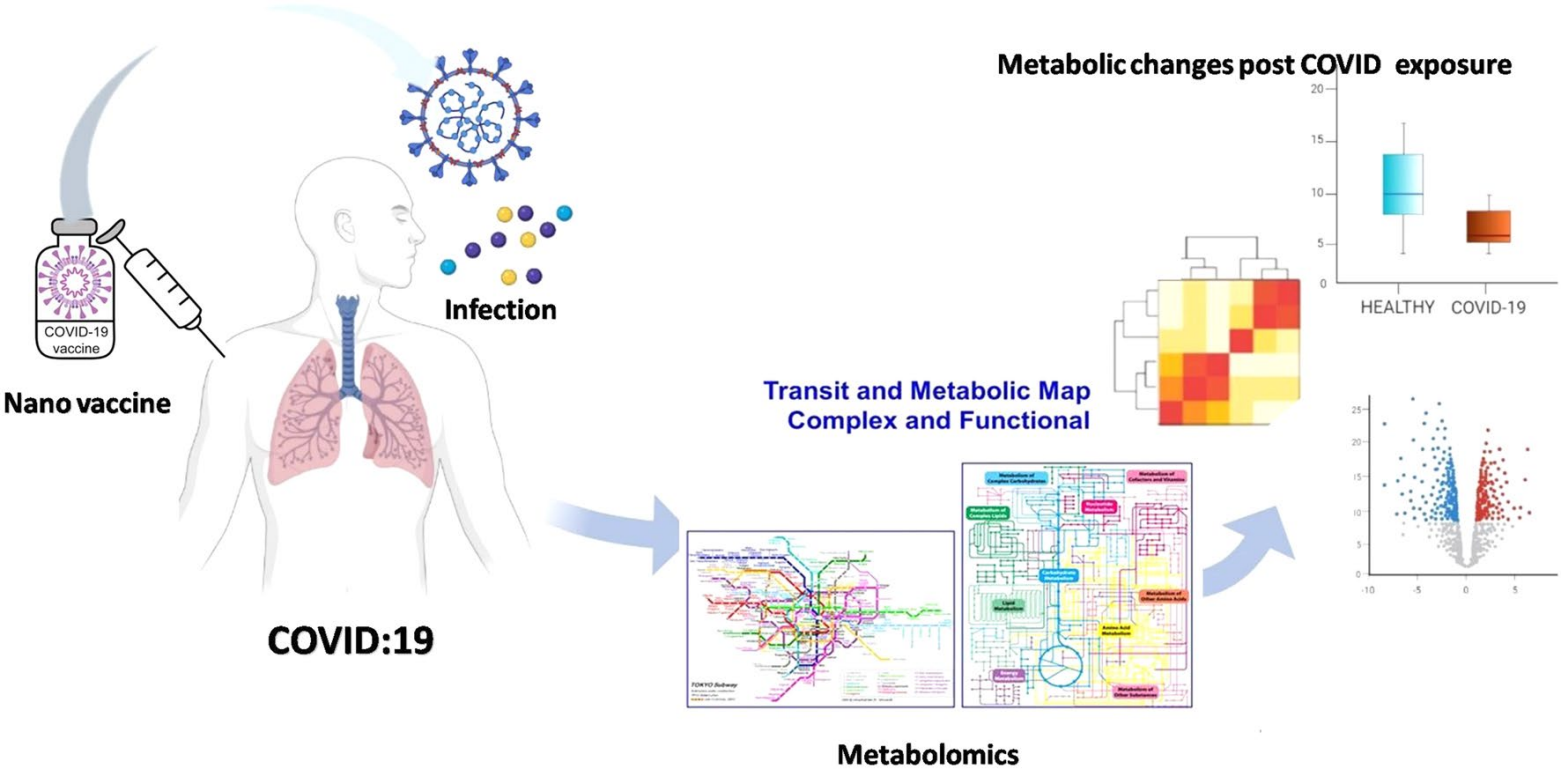

## Introduction

In December 2019, severe acute respiratory syndrome coronavirus 2 (SARS-CoV-2) was recognized as the causative agent of COVID-19 [1]. SARS-CoV-2 belongs to a prevalent class of viruses known as the β–coronaviruses [2]. Later, in March 2020 the World Health Organization proclaimed it as a global pandemic [1]. Hence, SARS-CoV-2 is currently regarded as the 21 century’s plague [2]. Being a highly contagious disease, COVID-19 poses a significant global threat to public health through its effects on the respiratory system with increased evidence for multisystem complications. The disease ranges from mild flu-like symptoms to acute fatal respiratory distress syndrome [3]. Such wide disparities in disease severity among different patients is probably caused by several underlying factors including genetic, and environmental vulnerabilities and associated comorbidities [4].

As a rapidly developing field of research, the use of metabolomics in the diagnosis of infectious diseases was given a boost by the COVID-19 pandemic [5]. Focused on phenotypic diversity, metabolomics can provide helpful mechanistic information for understanding distinctive responses to a specific disorder (COVID-19 for example) from different patients in comparison to normal cases [6–8]. Additionally, a detailed characterization of the metabolic readouts should facilitate the discovery of new therapeutic targets and biomarkers, of potential use in disease diagnosis and monitoring therapeutic activity [9]. Furthermore, analyzing the interrelation between metabolomics and immunity might potentially introduce new arsenals in our battle against several viral infections such as COVID-19. For instance, the novel SARS-CoV-2 variant Omicron S protein with a large number of mutations has shown a remarkable impact on the viral contagiousness and immune escape potential [10]. This is likely related to the development of SARS-CoV-2 specific T cell responses secondary to altered metabolite levels [11].

**Fig. 1:**
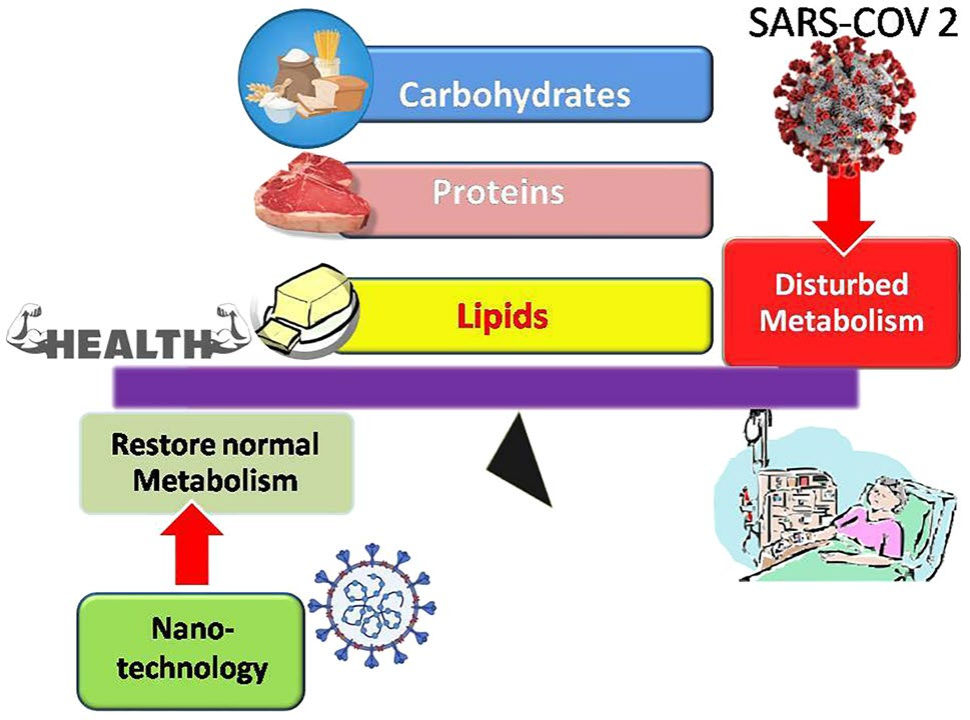
Integrated role of metabolomics and nanotechnology in COVID-19 management

Nanoparticles (NPs) represent a strategic answer to combat viral infections including coronaviruses such as SARS-CoV-2 [12]. First, they can be used for formulating vaccines as a protective measure against COVID-19 through cellular delivery of loaded cargo such as antigens [12]. This was achieved via vaccines based on mRNA technology delivered by lipid NPs [13]. Second, they can be utilized for treatment either through an inherent antiviral nature of certain nanomaterials or by acting as nanocarriers for antiviral agents. Nevertheless, NPs were increasingly reported to affect biological systems’ metabolome [14]. In this review, we focused on how exposure to COVID-19 and NPs can alter lipid, amino acid, and carbohydrate metabolism to illustrate what parameters to be considered when using NPs to combat viral infections in general and COVID-19 in particular. Figure 1 graphically depicts the proposed integrated role of metabolomics and nanotechnology against coronaviruses that will be described in this review.

## Search strategy

Literature review articles; formerly conceptualized as the ‘gold standard’, aim to update the guideline knowledge of pharmacologists, nutritionists and clinicians as well as guide their implementation into clinical practice [15]. A comprehensive literature search was conducted in this review using a rationalized search of the published literature with shedding light on the effect of nanotechnology and metabolomics in COVID-19 management. This comprehensive search was conducted on PubMed, Web of Science, ScienceDirect databases and Scopus electronic databases using database-specific search terms in two fields: COVID-19 metabolomics and Antiviral Nanoformulations. The selection criteria proceeded with the following key search terms: COVID-19; metabolome; metabolite; carbohydrate; lipid; protein; nanoformulation; nanotechnology; nanoparticle. The inclusion criteria were articles in the English language and articles with full texts mainly focusing on how COVID-19 and nanoformulations affect metabolite levels. Exclusion criteria included papers in languages other than English and papers with abstracts only. Data extraction followed a search strategy by compilation and comparison of all suggested articles to identify and select the recommended key terms. Finally, the reference lists of selected papers were also examined for recommended articles of relevance.

## Interrelationship between metabolomics and immunity in SARS-CoV-2 infection

### Lipid metabolism in COVID-19 patients

#### Steroids and triglycerides

Considering that viruses attack lipid signaling pathways for their survival, it is assumed that lipids play a crucial role in viral infection [16]. Emerging evidence suggests that lipid dysregulation may contribute to fatal complications correlated to COVID-19 disease severity [17]. Interestingly, previous studies reported that the accumulation of some steroid hormones such as androgens, estrogens and progesterone are associated with the modulation of macrophages besides other immune and non-immune cells in COVID-19 patients [18]. Additionally, serum levels of 21-hydroxypregnenolone, an intermediate for synthesizing corticosterone, increased in SARS-CoV-2 infected patients [18]. On the other hand, the sulfated form of dehydroepiandrosterone, DHEAS, an abundant steroid in human serum was found to be reduced in severe COVID-19 patients [19, 20]. Interestingly, male COVID-19 patients experienced a greater magnitude of DHEAS decline when compared with female patients [20]. This might explain why male COVID-19 patients have higher severity and mortality rates than female COVID-19 patients [21]. Depressed DHEAS and a higher cortisol/DHEAS ratio, have also been observed in aged people, critically ill patients, and those with inflammatory diseases [22]. It has been demonstrated that administration of DHEA supplements reduces pro-inflammatory cytokines and boosts regulatory T cells in inflammatory models [23]; that implies driving interest to investigate DHEA as a potential adjuvant in COVID-19 therapy.

COVID-19 patients have shown characteristic serum profile changes typical for severe dyslipidemia as compared to healthy controls [24]. Decrements in total cholesterol (TC) [24–26], TC-high-density lipoprotein (HDL) [25], TC-low-density lipoprotein (LDL) [25], apolipoprotein A2 (ApoA2) [26], ApoD [27], and ApoM [27] were reported in COVID-19 patients. Depressed ApoA1 protein levels in COVID-19 patients are negatively correlated with C-reactive proteins, IL-6, d-dimers, prothrombin time and thrombin time [28]. On the contrary, serum triglycerides (TG) [25, 26, 29], TG-HDL [25], TG-intermediate-density lipoprotein [24], TG-LDL [25], TG-very low-density lipoproteins (VLDL) [24, 30], and ApoB [24] increased. Elevated TG levels have been reported to diminish immunoglobulin G level, an antibody that protects against viral infections including the SARS-CoV-2 virus [31]. Interestingly, some authors reported the upregulation of VLDL levels for 6 months after COVID-19 infection [30], supporting the finding of altered lipid metabolism following SARS coronavirus infection [32]. Accumulation of TG and VLDL in COVID-19 patients is probably due to attenuated hepatic capacity to oxidize aceto-acetyl CoA inside the mitochondria with subsequent increase in ketone bodies like 3-hydroxybutyrate, acetoacetate, and acetone [25, 33].

#### Fatty and bile acids

Fatty acids represent the building blocks for viral replication. In addition, owing to their conversion to lipid mediators as eicosanoids, fatty acids are robustly involved in immune and inflammatory responses [34]. Arachidonic acid (AA) was recognized by system biology-based analysis as one of the key metabolic pathways mostly affected by COVID-19-infected cases [35]. It is assumed to have potent antiviral properties against enveloped viruses such as SARS-CoV-2 [36, 37]. However, its serum levels in COVID-19 patients were contradictory. Serum concentrations of AA were markedly decreased by 2-to-4-folds in COVID-19-positive patients compared to healthy controls [18, 38, 39]. Such decline was found to be associated with an increased state of systemic inflammation in diseased COVID-19 patients [40]. In contrast to these studies, elevated levels of AA, together with 5- and 11-hydroxyeicosatetraenoic acids were reported in the plasma of COVID-19 diseased subjects and found to strongly correlate with elevated acetylcholine concentration and likewise to disease severity [41]. This lipid/cholinergic mediator cross-talk was positively correlated to increased intensities of inflammatory and thrombotic markers, such as neutrophil to lymphocyte ratio, neutrophil counts, international normalized ratio, and cytokine levels (IL-1β, IL-6, and IL-8), thus contributing to COVID-19 immunopathology [41]. Regardless of increases or decreases in serum AA levels, it is postulated that once AA metabolites promote hyperinflammation and lethality in COVID-19 patients, it would be ineffective to use AA as a therapeutic tool [41]. Among the numerous protocols proposed as complementary treatments for COVID-19, the anti-inflammatory and immunomodulatory glucocorticoids have proven effectiveness; which is likely to be mediated through attenuation of both proinflammatory lipid mediators and acetylcholine levels [41–43].

Analysis of plasma levels of saturated fatty acids i.e., stearic, lauric, and palmitic acids showed a decrease in COVID-19 infection [38]. Such a decrease was postulated to be related to these fatty acids’ consumption during the activated biosynthesis of viral membrane phospholipids [38]. Alternative to diminished stores of poly-unsaturated fatty acids (PUFA), other metabolic transformations were found favored resulting in much higher levels of other fatty acids such as linoleic acid which showed 2- to 11-fold increase in COVID-19-infected intensive care unit patients [39]. Others include α-linolenic, nervonic, trans-vaccenic, and palmitoleic acids which positively correlated with a worse prognosis of COVID-19 [33]. Patients with severe and critical COVID-19 disease manifested modified expression of genes linked to fatty acids and derived bioactive lipid mediators’ pathways. That included upregulation of genes encoding proinflammatory proteins (OXER1, LTB4R) and downregulation of those encoding anti-inflammatory proteins (HACL1, THEM4) [41]. The product of the HACL1 gene has modulatory action on peroxisome proliferator-activated receptor alpha signaling with subsequent inhibition of hyper-inflammatory odd-chain fatty acids metabolites [44]. Meanwhile, the product of the THEM4 gene has been implicated in the anti-inflammatory actions mediated by vitamin D via inhibiting the production of lipid mediators derived from the cyclooxygenase-2 pathway [45].

As a consequence of altered fatty acid levels, oxylipins metabolism was also expected to be affected. Oxylipins are oxidation products of fatty acids and mediators of inflammatory responses of which levels tend to increase in oxidative stress [46]. Oxylipins are suggested to play a role in infection propagation, support the viral capsid membrane synthesis, targeting pro-inflammatory immune cells, and initiation of thromboembolic complications through platelet activation [47, 48]. COVID-19-infected intensive care unit patients displayed increased plasma levels of oxylipins derived from non-enzymatic peroxidation of PUFA which strongly correlated with markers of macrophage activation [39]. On the other hand, another study reported decreased serum levels of oxylipin products derived from the oxidation of AA and linoleic acid, namely 15-hydroxyeicosatetraenoic acid, and 9/13-hydroxyoctadecadienoic acids, respectively in COVID-19 patients [38]. This might be attributed to the deficiency of the precursor molecule AA, as previously discussed. High oxylipin levels in COVID-19-infected lung cells without associated elevation in the circulation were also disclosed in a third study [49]. A possible breakdown of high-density lipoproteins (HDL) by the antioxidant enzyme paraoxonase-1 during plasma transportation might account for the decreased or unaltered circulatory oxylipin levels [38, 50].

Acylcarnitines are specific markers for the β-oxidation of fatty acids, thus reflecting mitochondrial status. Elevated acylcarnitine levels result from incomplete fatty acids oxidation [51]. Impairment of mitochondrial β-oxidation of very long-chain and medium-chain fatty acids has been reported in several viral infections including that of COVID-19 [38, 52]. Several studies showed an increase in acylcarnitines serum levels in positive COVID-19 patients versus a decrease at hospital discharge [33, 53]. This is in line with the finding of acylcarnitines accumulation in respiratory viruses such as influenza virus, where they co-localize with pulmonary surfactant with subsequent reduction of surface tension and prevention of alveolar collapse during breathing [54]. Contrary to these findings, other studies reported a decrease in acylcarnitine serum levels in COVID-19 patients [48, 55], which might translate into a defective anticoagulant function [56], and in consistence with the hypercoagulable phenotype in COVID-19 patients. Such variability in acylcarnitine levels should encourage more analysis from different race groups to be conclusive.

Besides their role in cholesterol elimination and enhanced absorption of fat-soluble nutrients, bile acids act as signaling molecules either to boost or hamper virus replication [57] that has been demonstrated in different viruses including herpes simplex [58], influenza A [59], hepatitis B and C viruses [60]. Involvement of bile acids signaling in SARS-CoV2 viral replication is possible but still needs investigation of proof of concept. Bile acid metabolites such as taurocholic acid, taurodeoxycholic acid, glycodeoxycholic acid, glycocholic, and glycoursodeoxycholic acid were found to increase with the severity of COVID-19 infection [33]. In other studies, secondary bile acids such as deoxycholic acid and ursodeoxycholic/hydrodeoxycholic acid showed lower levels in COVID-19 patients [38, 61].

#### Phospholipids and sphingolipids

Studies have pointed out altered levels of phospholipids in COVID-19 infection. Choline (a common component of most phospholipids) was reported to decrease specifically in severe COVID-19 cases. Meanwhile, a significant increase in phosphocholine (an intermediate in phosphatidylcholine synthesis) was observed [18, 19]. This could be likely attributed to the increased polarization of macrophages in response to viral infection leading to an augmented absorption of choline required for phosphocholine formation which in turn promotes elevated cytokine secretion [62]. Besides, increased phosphocholine stimulates phagocytosis and endocytosis [63]. Contradictory results were observed in phosphatidylcholine (PC) and lysophosphatidylcholine (LPC) levels in COVID-19 cases. Decreases in both PC and LPC plasma levels were reported in COVID-19-positive patients [64, 65]. Other studies showed decreases in PC versus increases in LPC [53, 55, 60] or even the other way round where PC was increased [38, 67] and LPC was decreased [33, 38, 68, 69]. Such discrepancy in the observed effects might be attributed to differences in the characteristics of the studied patient groups [38]. Supporting this hypothesis, another recent study reported opposing phospholipid profiles in the plasma of two subgroups of COVID-19-diseased subjects [70]. They speculated such variability to be probably related to an interplay between inflammation and thrombosis with systemic oxidative stress and altered immune system being potential predictors of possible lethal outcomes [70]. In cellular models, an exploratory functional evaluation of LPC 16:1 and lysophosphatidylethanolamine18:1 revealed their capacity to induce membrane disruption, elevate intracellular calcium and cytokines, and mediate apoptosis [71]. Thus, indicating the possible use of particular phospholipids with functional impacts in assessing the severity and pathogenesis of COVID-19 with consequent clinical decision-making [71].

Another class of potent structural bioactive lipids which mostly exist in nervous tissues is sphingolipids. They have been implicated to play a role in the pathogenesis of several respiratory tract infections through the regulation of inflammatory processes and in the modulation of host–pathogen interactions [35, 72]. Ceramides and sphingosine-1-phosphate (S1P) are central metabolites of sphingolipids with crucial roles in the control of immune cell activation, trafficking, and inflammation [73, 74]. Studies on COVID-19 patients revealed elevated sphingolipids levels [19, 35]. Another study reported depressed sphingolipids and glycerophospholipids levels both in non-severe and severe COVID-19 patients which were assumed to result from liver damage [18]. In a lipidomic analysis of the plasma of COVID-19-infected individuals, total levels of ceramides showed an increase of more than 250-fold in patients with mild symptoms [75]. Those with severe respiratory distress symptoms showed a total ceramide level increase of more than 450-fold, thus suggesting a potential role of ceramides in COVID-19-associated respiratory disease [75]. On the other side, circulatory S1P levels act as a biomarker of the severity and mortality of COVID-19 [76], where restoring S1P levels has been advised to be a promising COVID-19 therapeutic target [77].

In favor of the potential therapeutic roles played by lipids in human health, a transcriptomic and broadly targeted lipidomic approach has identified, and pharmacologically evaluated novel lipid compounds from the rind of sugarcane against SARS-COV-2 [78]. Interestingly, 2-linoleoylglycerol and gingerglycolipid C have displayed strong binding interactions with Cys145 and His41 residues of the main protease 3CL^pro^ of SARS-CoV-2 in molecular docking studies [78]. The 3CL^pro^ is essential for SARS-CoV-2 replication [79], therefore, inhibiting 3CL^pro^ using these lipids poses them as promising therapeutic candidates [78].

### Amino acids/biogenic amines metabolism in COVID-19 patients

#### Tryptophan-kynurenine pathway

Widespread dysregulation of amino acids and biogenic amine metabolism has been also detected in COVID-19 patients. Pro-inflammatory signaling in COVID-19 favors proteolysis and amino acids catabolism as evidenced by changed levels of several amino acids, together with elevated oxidative stress, and inflammatory markers. Several studies have supported the decreased levels of tryptophan, an important regulator of inflammation and immunity in COVID-19 patients [33, 48, 68, 80–82]. Kynurenine, an important immunosuppressive metabolite of tryptophan, and its downstream product kynurenic acid displayed significant increases in COVID-19 patients [18, 33, 48, 68, 80, 81]. Increased kynurenine/tryptophan, and kynurenic acid/kynurenine ratios were found positively correlated with inflammatory cytokines and poor prognosis of COVID-19 infection [35, 48, 68, 83, 84]. A positive correlation between anthranilic acid level, a product of the kynurenine pathway and maintenance of elevated IL-10, and IL-18 levels were reported [81]. Moreover, dysregulated tryptophan metabolism was reported in many inflammatory conditions ultimately leading to decreased synthesis of nicotinamide adenine dinucleotide known as NAD^+^ as it is synthesized from tryptophan by the kynurenine pathway [85]**.** Hence, being a cofactor in many cellular redox reactions, NAD^+^ can function as a switch for macrophage effector response [86] as in the case of viral infections. Altogether, this supports previous reports on the modulatory role played by the tryptophan-kynurenine pathway on T-cells and macrophage-mediated responses [86–90]. Thus, confirming the key contribution of this pathway to COVID-19 infection.

#### Urea and tricarboxylic acid cycles

Another two disordered metabolic pathways in COVID-19 infection are the urea and tricarboxylic acid (TCA) cycles which are in control of amino acids catabolism, and energy metabolism. Metabolites belonging to or closely related to these two cycles showed significant changes. Arginine, a crucial amino acid of the urea cycle, was found markedly decreased in severe COVID-19 patients as compared to healthy controls [18, 81, 91]. On the other hand, an increase in arginine levels in critical care COVID-19 patients was found [48, 81], or even a lack of change in arginine levels between acute and recovery phases of COVID-19 patients was also reported [80]. Serum levels of arginine derivatives like asymmetric dimethylarginine (ADMA), symmetric dimethylarginine (SDMA), homoarginine, and *N*-acetylarginine were found to decline in non-severe COVID-19 patients, hence suggesting hepatic dysfunction [18]. Urea cycle intermediates involved in arginine catabolism namely, ornithine, and citrulline displayed either declined levels [18, 48, 80], or elevated levels [19, 91] in COVID-19 patients. Moreover, significant ornithine and citrulline level differences could be observed between the acute and recovery phases of COVID-19 patients [80]. A highly significant correlation between ornithine and cytokine storm and coagulation index was described [92]. Polyamines such as spermine, and spermidine, and their mono-or-diacetylated derivatives were found to increase, both in non-cancer [19, 81] and cancer [93] COVID-19 patients, suggesting an amplified polyamine biosynthesis from arginine. Among acetylated polyamines, N1-acetylputrescine showed a correlation with cytokines as interferon α2a (IFNα2a), IFNγ, IL-2, and IL-10 [93]. The fact that arginine can be further metabolized to creatine and creatinine suggested the implication of arginine catabolism in disturbed kidney functions in COVID-19 patients [80, 94]. This was supported by the elevated creatine and creatinine levels that were observed in COVID-19 patients and were reported to positively correlate with inflammatory cytokines expression [48, 91]. An important amino acid intermediate in the TCA cycle, glutamine has shown reduced levels both in mild and severe COVID-19 patients [18, 35, 80, 95] with a tendency to increase back in recovered COVID-19 patients [30]. Such alteration was found to negatively correlate with C-reactive protein, lactate dehydrogenase, and partial oxygen pressure and to positively correlate with partial carbon dioxide pressure, thus promoting disordered oxygen hemostasis and lung damage in COVID-19 patients [95]. Furthermore, a deficiency of glutamine might inhibit M2 macrophage polarization as a compensatory mechanism, contributing to the hyper-inflammatory response observed in severe COVID-19 cases [80]. Nevertheless, considering that glutamine serves as a hub metabolite in several metabolic pathways, regulation of its production in COVID-19 patient needs further studies ideally using isotopomer-based metabolomics to discern its different pathways [96]. Another TCA intermediate, 2-oxoglutarate displayed an increase with the highest levels in severe COVID-19 patients, suggesting a seriously affected TCA cycle [91]. In contrast, a previous study on COVID-19 patients reported decreased sera levels of the amino acids [18].

#### Amino acids

In severe COVID-19 patients, serum phenylalanine levels were massively elevated by 100% [97]. This finding came to support other studies suggesting that increased phenylalanine levels can function as a potential marker for disease severity in COVID-19-infected patients [19, 25, 80, 81, 95, 98]. Elevated plasma levels of phenylalanine are further translated into lower tyrosine levels indicating a disturbed immune system owing to the induction of apoptosis as observed in human B-cells, thus facilitating viral infection and the attack of opportunistic pathogens [97, 99–101]. Moreover, higher phenylalanine levels signify an enhanced catabolic state where inflammatory cytokines induce muscle breakdown, releasing phenylalanine for gluconeogenesis to nourish the metabolic needs during COVID infection [98]. Nevertheless, one study suggested phenylalanine to be a distinct marker of COVID-19 disease severity with an independent correlation to both the onset of symptoms and the magnitude of inflammatory status [98]. A correlation analysis between different amino acids and inflammatory markers in COVID-19 patients was examined in several studies. Amino acids such as asparagine, isoleucine, leucine, and valine displayed a positive correlation with levels of tumor necrosis factor (TNF-α), proline with IL-17, and threonine with IL-26 levels [19]. Others highlighted an elevated level of acetyl-methionine and hydroxyproline in COVID-19 sera, which translates to defective proteolysis and collagen catabolism in these patients [48]. Such dysregulation might be attributed to the activated pro-inflammatory state being observed among COVID-19 patients as these pro-inflammatory signaling pathways prefers amino acids catabolism and proteolysis [102]. Likewise, studies showed elevated serum levels of methionine sulfoxide and cystine, along with decreased sulfur-containing amino acids (cysteine and taurine) which latter acts as antioxidants [103].

#### Neurotransmitters

Studies have also addressed variations in neurotransmitters levels in COVID-19 infection. Serum levels of glutamate were found elevated by 33% [25] and 22% [97] in COVID-19 patients. Other reports supported such upregulation in moderate and severe COVID-19 patients [19] and recovered patients as well [30]. On the contrary, a reduction in glutamate and *N*-acetyl-l-glutamate levels were observed in one study [18]. Catabolism of glutamate was also defective in SARS-CoV-2-infected patients resulting in the reduction of derived amino acids such as serine, aspartate, alanine, proline, and tyrosine [35, 80]. According to Shen and co-workers, levels of gamma amino butyric acid (GABA) showed a decline in consistency with its precursor glutamate [18]. It has been postulated that SARS-CoV-2-induced downregulation of angiotensin-converting enzyme 2 (ACE2) expression is linked to disturbed serotonin and dopamine pathways [104, 105]. Such a hypothesis was based on gene co-expression, co-regulation, and function similarities between ACE2 and DOPA decarboxylase, an enzyme involved in the biosynthesis of serotonin, dopamine, and histamine [105, 106]. Serotonin levels were found to be reduced by 2- and 3-folds in non-severe, and severe COVID-19 patients, respectively, as compared to healthy controls [18]. Other groups supported the downregulation of serotonin [19, 48, 84] with an inverse correlation to IL-6 levels [84] and platelet count [107]. It has been shown that increasing serum levels of serotonin might exert a potential antiviral effect through modulating the respiratory symptoms, and potentiating immune response with significant elevation in antioxidant properties and immunoregulatory effects [108]. Fluctuations in the levels of epinephrine, a metabolite of tyrosine, might be related to the severity of COVID-19. On one hand, it was assumed that the difference in COVID-19 severity between children and adults, being less severe at a young age is partially related to their greater fluctuations in epinephrine levels [109, 110]. It was shown that the proliferation of CD8+ T cells was positively correlated to epinephrine changes with a tendency to increase following epinephrine infusion [110, 111]. On the other hand, epinephrine was found to initiate and fuel the cytokine storms in lipopolysaccharide-treated mice and in a different model system of severe infection, resulting in exacerbation of the disease course [112, 113]. This suggests a possible contribution of epinephrine in worsening cytokine storm in COVID-19 patients [114].

### Carbohydrates/sugars metabolism in COVID-19 patients

Elevated glucose circulating levels consistent with altered carbon homeostasis, were found to correlate with inflammation markers in COVID-19 patients [48]. A glucose shift has been linked to increased release of cytokines TNF-α, IL-6, and IL-1β during host cell viral entry, replication, and exit [97, 115]. This accounts for the observation that diabetic patients having uncontrolled glucose levels are more susceptible to severe SARS-CoV-2 infection [116, 117]. An increase in glucose levels by 68% [25] and 83% [97] in the serum of COVID-19 patients was observed. Furthermore, Shen and co-workers reported higher glucose and glucuronate levels in COVID-19 patients [18], likely explained by the elevated glycolytic effect of monocytes and macrophages during SARS-CoV-2 infection, thus mediating viral replication [118]. Moreover, SARS-CoV-2 infection augments the production of mitochondrial reactive oxygen species (ROS) with subsequent stabilization of hypoxia-inducible factor-1α (HIF-1α) and consequently induced glycolysis [118]. HIF-1α also affects monocyte metabolism, causing direct suppression of T-cell response and reduced survival of lung epithelial cells [118]. Blocking glycolysis combated SARS-CoV-2 infection, where it attenuated its replication in Caco-2 cells [119].

Under anaerobic conditions, typical for COVID-19, glucose proceeds through glycolysis and gets fermented to lactate producing a restricted amount of adenosine triphosphate (ATP), thereby resulting in elevated blood lactate and lactate dehydrogenase levels [19, 91, 97, 121, 122]. Meanwhile, high replication of SARS-CoV-2 viruses is linked to accelerated consumption of ATP depleting it, which suppresses vital metabolic processes i.e., pentose phosphate pathway, blood glucose uptake, oxidative decarboxylation of pyruvate and TCA cycle [95, 97, 119, 120, 123].

**Fig. 2:**
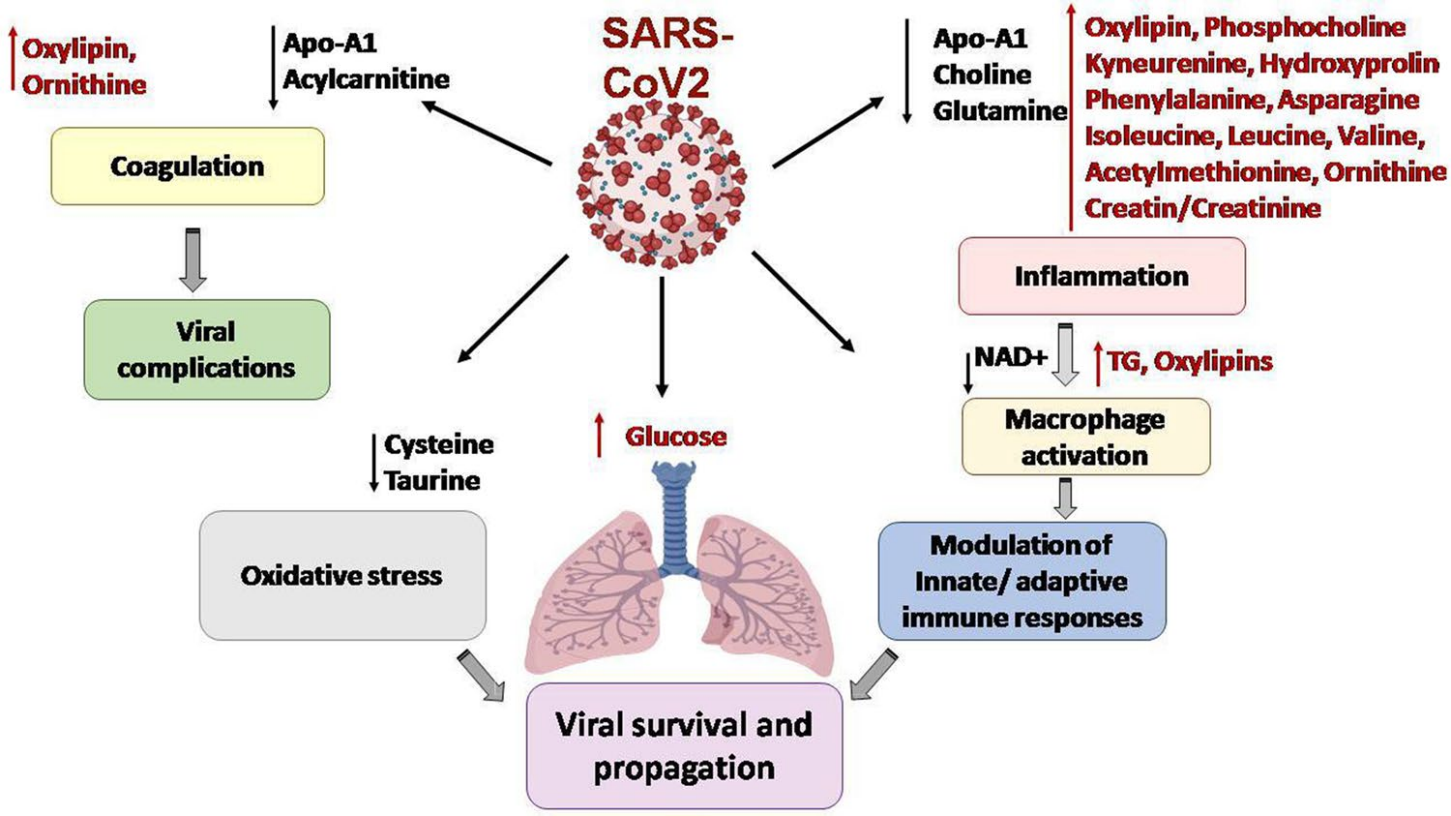
Metabolic dysfunctions observed in COVID-19

**Fig. 3:**
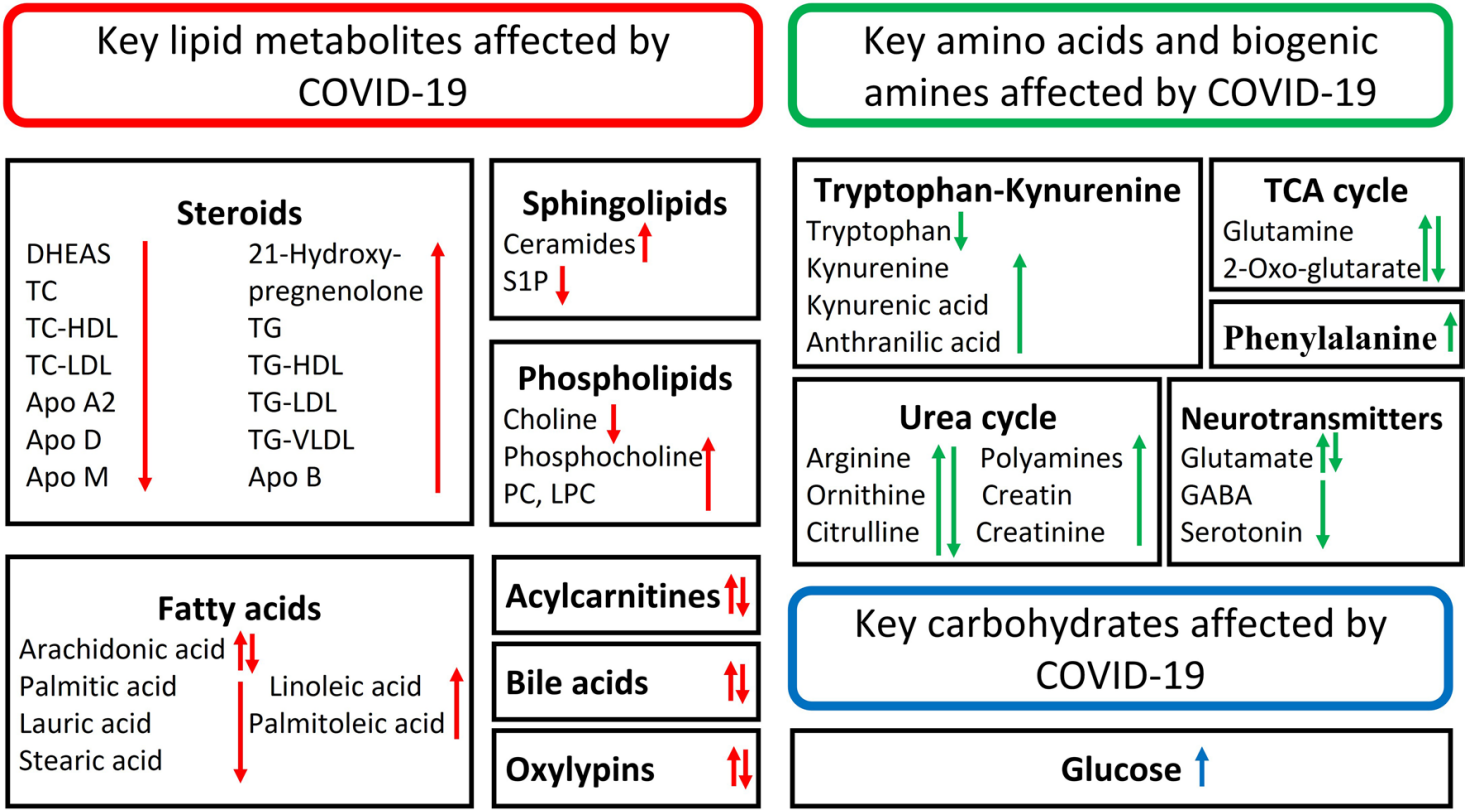
Key metabolites affected by COVID-19. ↑: indicate increases, ↓: indicate decreases, ↑↓: indicate contradictory findings. *DHEAS* dehydroepiandrosteronesulphate, *TG* triglycerides, *TC* total cholesterol, *HDL* high-density lipoprotein, *LDL* low-density lipoproteins, *VLDL* very-low-density lipoprotein, *S1P* sphingosine-1-phosphate, *PC* phosphatidyl choline, *LPC* lysophosphatidylcholine, *TCA* tricarboxylic acid, *GABA* gamma amino butyric acid

It is noteworthy to mention that the variability in COVID-19-mediated metabolic signatures perturbations are consequent not only to the disease stages (mild, moderate, severe, critical or recovery) or to interpatient factors but also is undeniably a matter of the pandemic wave from which samples have been collected [124]. Based on the aforementioned observations, it can be concluded that targeting the dysregulated metabolic pathways and focusing on the altered metabolites might be an optimum therapeutic approach for COVID-19 management as illustrated in Fig. 2. A collective summary of the key metabolites affected by COVID-19 is illustrated in Fig. 3.

## Nanoparticles as antiviral agents

Nanoparticles include particles of size range 1–1000 nm. According to the material of construction, they can be classified into inorganic and organic NPs [125]. Inorganic NPs comprise, for example, gold, silver, zinc oxide and titanium dioxide NPs [126]. While organic NPs can be divided into polymeric and lipidic NPs. Polymeric NPs refer to particles formulated from polymers such as poly(lactic-co-glycolic acid), poly(lactic acid) and others **(**[127]. Lipidic NPs designate particles made of lipids such as phospholipids, mono-, di-, triglycerides, fatty acids, and others [128]. These versatile NPs were found to possess potential biological activities such as anticancer, antioxidant, immunoprotective and antimicrobial attributes [129–133]. Additionally, they are well-documented to treat viral infections either on their own [133, 134] or as carriers loaded with drugs [135]. This can be attributed to their improved physicochemical properties (nano-size, charge and shape), feasibility, cost-effectiveness, tenability, non-toxicity, biocompatibility and multi-functionalities [136]. Their tailored high surface-to-volume ratio and optical and functional characteristics may be the basis of these critical properties accounting for NPs’ antiviral effects. Moreover, NPs have the potential to cross cell membranes and enter the cell, facilitating their interaction with sub-cellular structures [137]. Indeed, the progression of viruses̛ resistance to conventional antiviral agents leads to a continuous demand for discovering alternative treatments such as those based on nanotechnology [138].

**Fig. 4:**
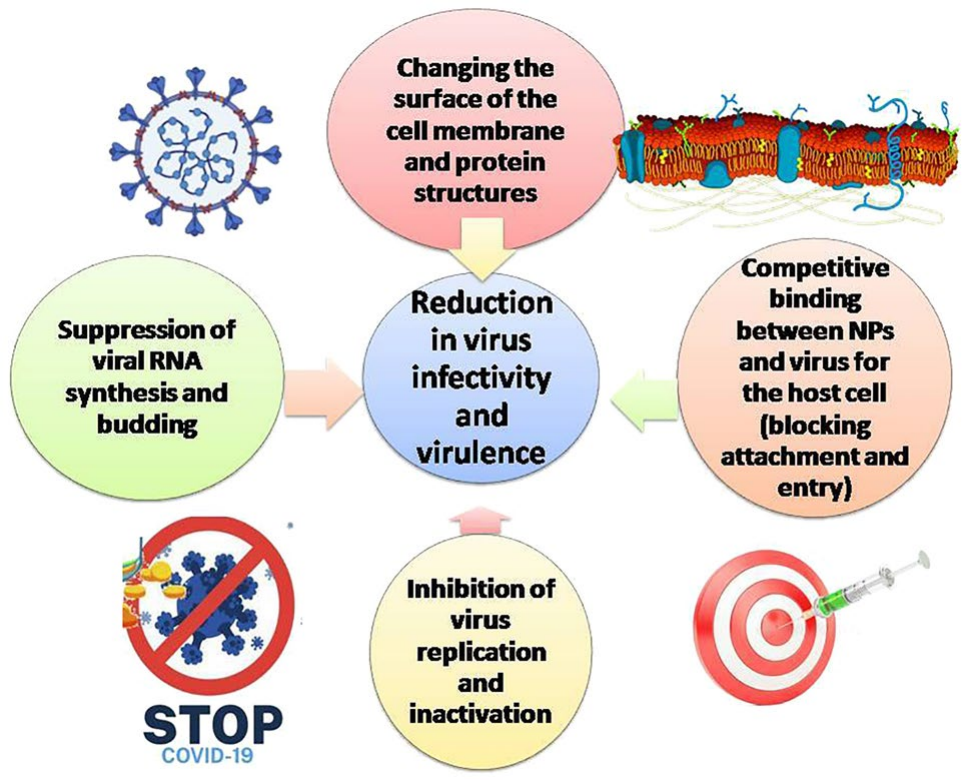
Role of antiviral nanoparticles in abolishing viruses’ infectivity and virulence

The role of NPs in viral therapeutics is not limited to only one specific type but rather to several types that showed promising therapeutic outcomes against different viruses such as gold NPs [139, 140], silver NPs [141–144]**,** zinc oxide (ZnO) NPs [145, 146], titanium dioxide (TiO_2_) NPs [147, 148], and selenium NPs [149, 150]. Although the antiviral effects of NPs were investigated against a variety of viruses, their exact mode(s) of antiviral action are still not determined. Several reports analyzed the antiviral effects of NPs, focusing on their possible influence on virus binding to host cells, penetration, replication of viral genomes and budding [133, 151–153]**,** hence suggesting possible mechanisms as presented in Fig. 4**.** NPs can interfere with the virus attachment to host cells such as the antiviral mechanism of gold [154] and silver NPs [140] against herpes simplex virus. Moreover, silver NPs can bind to viral surface proteins, owing to their small size and large surface area, preventing interactions with cells [144]**.** Another mechanism is the induction of ROS, which damages viral particles [134]**.** Cationic polymers and oligomers have demonstrated such antiviral activity [155]. Moreover, silicon NPs interfered with virus replication by reducing the number of produced viruses from cells infected with influenza A virus [156]**.**

Notably, NPs present a paramount promise against coronaviruses e.g., severe acute respiratory syndrome (SARS) and Middle East respiratory syndrome (MERS) viruses [1, 151, 157–159]. For example, Du and colleagues investigated the mechanistic role of silver-coated gold nanoparticles on the long-term inhibition of the replication cycle of the Porcine epidemic diarrhea virus (PEDV); a virus model of the Coronavirus family [160]. Plaque assay and visualization of infected cells conducted in this study revealed a decrease in plaque-forming units and viral titer as well as the cytopathic effect via pre-treatment with NPs. The developed NPs shared the capacity to block PEDV cellular entry and suppress mitochondrial membrane potential and caspase-3-mediated apoptosis. Against SARS-CoV-2, NPs proved their efficacy in vaccination and showed potential in treatment. Using lipid NPs to deliver mRNA-encoding virus proteins represented a breakthrough in the world’s fight against COVID-19 and saved thousands if not millions of lives. Regarding treatment, silver NPs are of particular interest as they displayed a potential against respiratory viruses including SARS-CoV-2 [161]. Silver NPs with size 10 nm successfully disrupted in vitro SARS-CoV-2 viral integrity and hindered its entry step into cells [162]. Furthermore, silver NPs with different sizes and surface modifications inhibited the activity of SARS-CoV-2 in Vero E6 cells, where NPs coated with polyvinylpyrrolidone or branched polyethyleneimine were found more potent than those coated with citrate [163]. Additionally, a nano-immunotherapy based on a nanostructured inorganic phosphate complex attached to a glycoside protein termed OncoThread^®^ was found to exert a beneficial effect in SARS-CoV-2 management. Through its immunomodulatory action, it stimulated the immune system and reduced pulmonary inflammation and shortened the hospitalization period for COVID-19 patients [164].

## Nanoparticles-associated changes in the cellular metabolome

Besides investigating the clinical effectiveness of NPs as antiviral agents or carriers, it is equally important that studies should take into consideration issues related to their nanotoxicological profile on animals and humans. Indeed, NPs could interact with various immune cells of biological systems [165] and impact metabolite levels inside the body and cells [14, 166]. Such changes in metabolism could lead to their adversity for the nominated COVID-19 fighters.

Metabolomics analysis, typically defined as the untargeted analysis of metabolome in biological systems, is increasingly applied as a crucial assessment of NPs’ biological toxicity via performing metabolic profiling using mass spectroscopy and/or nuclear magnetic resonance [166, 167]. Metabolomics analysis post-exposure to drug treatment has been increasingly applied to assess drug safety based on the analysis of a large set of metabolites in examined cells or animal models [168]. For example, BioNTech/Pfizer (BNT162b2) vaccine based on mRNA technology downregulated TC, TC-LDL, phospholipids, Apo B100, VLDL-phospholipids and TG in vaccinated subjects without prior infection with COVID-19. While previously infected then vaccinated individuals showed minor changes [169]. Moreover, mRNA COVID-19 vaccination increased levels of certain amino acids namely 3-methylhistidine l-histidine, l-glutamine, and l-phenylalanine [170].

### Gold nanoparticles

Gold NPs affected lipid and glucose metabolism in obese mice, as manifested by normalized glycemic control and significantly decreased plasma non-esterified fatty acids. Additionally, they upregulated macrophage (F4/80) and pro-inflammatory markers (TNFα, toll-like receptor-4 (TLR-4)) expression in the fat tissue, irrespective of NPs’ dose regimen. The opposite occurred in the liver tissue, where gold NPs downregulated macrophage (F4/80) and expression of inflammatory markers (TNFα and TLR-4) [171]. Moreover, PEGylated gold NPs injected in rats increased serum triglycerides and cholesterol levels [172]. Protein metabolism was also disrupted by gold NPs, where proteins associated with cellular oxidative stress and related to gluconeogenesis (phosphoenolpyruvate carboxykinase 2) and cytoskeleton (actins and tubulins) increased in fish. This effect was more pronounced in the case of citrate-coated gold NPs than polyvinylpyrrolidone-coated ones [173]. Furthermore, human dermal fibroblasts exposed to gold NPs increased glutathione level concurrent with enhanced cellular protection from oxidative stress and consequently cytotoxicity [166]. Gold NPs coated with polyvinylpyrrolidone significantly increased inosinic acid, NAD, and guanosine monophosphate (GMP) in phagocytes isolated from sea urchins. While sialic acid and sulfated metabolites were significantly decreased in comparison to control cells. These changes suggested an increase in the phagocytic activity with a metabolic shift towards resolving an inflammatory response [174].

### Silver nanoparticles

Silver NPs perturbed carbohydrates metabolism as manifested by a reduction in lactate release and glucose consumption in hepatoma cells. Additionally, they reduced the expression of pentose phosphate pathway regulatory molecule; nuclear factor erythroid 2-like 2 (Nrf2) [175]. Moreover, silver NPs affected the TCA cycle in human dermal fibroblasts. They elevated citric acid levels which in turn reduced the biosynthesis of malic acid leading to cytotoxicity [166]. Coated silver NPs with polyvinylpyrrolidone affected protein metabolism by raising levels of glutamine, glutamate, glycine, and methionine in mice lungs, suggestive of de novo glutathione biosynthesis [176]. Furthermore, silver NPs increased triiodothyronine (T3) levels in hens depending on the size and concentration of NPs taken orally. However, serum steroid hormones and thyroxine (T4) levels were not affected [177].

### Zinc oxide nanoparticles

ZnO NPs are known for their hypoglycemic effect, where they lowered blood glucose in hens [178]. Additionally, they lowered blood glucose in diabetic mice by 40% at a dose of 14 mg/kg [179]. Similarly in diabetic rats, they reduced glucose levels by 56% and elevated insulin levels by 93% at a dose of 5 mg/kg for 15 days [180]. This hypoglycemic effect was even potentiated when ZnO NPs were combined with chromium oxide and selenium nanoparticles [181]. However, at higher doses an opposite response was reported, where hyperglycemia occurred. A dose of 25 mg/kg increased plasma glucose levels in mice due to the development of insulin resistance through the induction of endoplasmic reticulum stress [182]. ZnO NPs also affected lipid metabolism by lowering serum triacylglycerol in quails [183] and diabetic rats [181]. Additionally, in lactating mice they induced fatty acids biosynthesis, glycolysis, and glutathione metabolism by 3.5, 3.6 and 4.4 folds, respectively. On the other hand, they reduced the production of milk fat by 51.8% indicating lower lactogenesis and suggesting oxidative stress in the mammary glands [68]. Contrarily, they demonstrated an antioxidant effect by upregulating mRNA levels of antioxidant enzymes such as superoxide dismutase, catalase, and glutathione peroxidase in quails [183] suggestive of more studies in different models for a conclusive effect on lactation. Protein metabolism was also affected by ZnO NPs as manifested by increased levels of valine and isoleucine as essential branched amino acids in hens after adding ZnO NPs to their diet suggesting an increase in protein assimilation [178].

### Titanium dioxide nanoparticles

TiO_2_ NPs perturbed lipid metabolism by lowering serum TG, increasing lipid peroxidation marker malondialdehyde with no effect on serum TC, TC-HDL, and TC-LDL levels in rats [184]. Moreover, they significantly changed levels of sphingolipids, prenol lipids, fatty acyls and glycerophospholipids in human bronchial epithelial cells (BEAS-2B). In addition to promoting steroid biosynthesis and upregulating cholesterol synthesis pathways. TiO_2_ NPs were suggested to cause oxidative stress in cells which resulted in the observed metabolic disturbances [185]**.** Furthermore, TiO_2_ NPs disrupted carbohydrate metabolism by inducing hyperglycemia in mice at doses of 50 mg/kg or higher [186]. This effect was even more pronounced in younger animals compared to adults and was attributed to induced insulin resistance through hepatotoxicity [187]. Moreover, they showed signs of oxidative stress by reducing lactogenesis and milk fat production by 35.7% in the mammary glands of lactating mice [188]. Similarly in rats, TiO_2_ NPs raised levels of acetylornithine, methionine sulfoxide and non-essential amino acids suggesting minor systemic oxidative stress [189].

### Other nanoparticles

Vanadium pentoxide nanoparticles (V_2_O_5_) affected both lipid and protein metabolism in human bronchial epithelial cells (BEAS-2B). They upregulated fatty acid oxidation and de novo biosynthesis indicating early signs of pulmonary fibrosis, in addition to upregulating amino sugars and downregulating glycosphingolipid metabolism [190]**.** Similarly, cerium dioxide (CeO_2_) NPs showed signs of inducing lung fibrosis in human bronchial epithelial cells (BEAS-2B), which was also validated in mice [191]**.** They altered lipid metabolism, particularly the S1P pathway and fatty acid oxidation. They increased expression of sphingosine kinase 1 increasing levels of S1P and its metabolites. Also, they increased metabolites resulting from the oxidation of fatty acids such as acylcarnitines.

Silica NPs reduced glutathione levels, whereas they elevated ROS and malondialdehyde levels in mice’s livers indicating oxidative stress. Furthermore, they disrupted protein metabolism downregulating three main pathways: (1) alanine, aspartate, and glutamate, (2) arginine and proline and (3) glycine, serine, and threonine [192]**.** Polymeric NPs formed from poly(lactic-co-glycolic acid) (PLGA) either uncoated or poloxamer-coated caused oxidative stress in macrophages in vitro. They interfered with protein metabolism by increasing the levels of arginine, proline, and N4-acetylaminobutanal. Furthermore, they affected cell membrane integrity by reducing levels of PC and phosphatidylethanolamines, which are essential membrane components. On the contrary, PEGylated PLGA NPs showed less disturbance in amino acid metabolism and cell membrane’s glycerophospholipids composition [193]. A summary of the effect of versatile NPs on various metabolites and metabolic pathways is provided in Table 1.

**Table 1:** Effect of different types of nanoparticles on metabolomics

Type of nanoparticles	Dose administered	Effect on metabolomics	References
Gold nanoparticles	0.0785 µg/g/day 0.785 µg/g/day 7.85 µg/g/day IP for 5 weeks in mice	Affected lipid metabolism by decreasing plasma non-esterified fatty acids and increasing plasma high-density lipoprotein cholesterol with no effect on plasma triglycerides	[171]
	0.7 mg/g single IV injection in rats	Affected lipid metabolism by increasing serum triglycerides and cholesterol levels	[172]
	80 µg/l in artificial sea water of fish	Increased proteins associated with cellular oxidative stress, gluconeogenesis, and cytoskeleton	[173]
	200 µM incubated with human dermal fibroblasts for 4, 8, 24 and 72 h	Increased glutathione levels protecting from cytotoxicity	[166]
	0.1, 1 and 10 µg/ml incubated for 24 h with immune cells isolated from sea urchins	Increased nicotinamide adenine dinucleotide and inosinic acid Decreased sialic acid and sulfated metabolites	[174]
Silver nanoparticles	Different hepatoma cell lines were treated with 0.5–7.5 µg/ml for 3 h	Affected glucose metabolism by reducing glucose consumption and lactate release in cells	[175]
	200 µM incubated with human dermal fibroblasts for 4, 8, 24 and 72 h	Affected tricarboxylic acid cycle elevating citric levels and reducing malic acid biosynthesis	[166]
	3 mg/kg BW through intratracheal instillation in mice	Affected protein metabolism elevating levels of glutamine, glutamate, glycine, and methionine in mice lungs	[176]
	1 ml/kg BW of hens with the following concentrations 1 ppm, 10 ppm and 100 ppm	Affected steroid hormones increasing triiodothyronine (T3) but not thyroxine (T4) levels	[177]
Zinc oxide nanoparticles	10 mg/kg BW added to feed of hens for short (4 weeks) and long exposure (24 weeks)	Lowered blood glucose levels Affected protein metabolism by increasing levels of valine and isoleucine	[178]
	Oral 8 and 14 mg/kg BW to healthy mice Oral 14 mg/kg BW to diabetic mice for 14 days	In healthy mice reduced blood glucose level by 25% (8 mg/kg BW) and 29% (14 mg/kg BW) In diabetic mice reduced blood glucose by 40% (14 mg/kg BW)	[179]
	Oral 5 mg/kg BW to diabetic rats for 15 days	Reduced glucose levels by 56% and elevated insulin levels by 93%	[180]
	10 mg/kg BW to diabetic rats for 6 weeks	Reduced blood glucose levels by 2.9-fold and increased insulin levels by 1.7-fold Affected lipid metabolism by lowering serum triacylglycerols	[181]
	25 mg/kg BW orally administered to mice	Increased blood glucose levels	[182]
	15, 30 and 60 mg/kg BW to Japanese quails for 60 days	Affected lipid metabolism by lowering serum triacylglycerol	[183]
	0.2 mmol/kg BW nasally administered to lactating mice from lactating days 1 to 12	Reduced milk fat production by 52% and induced fatty acids biosynthesis, glycolysis, and glutathione metabolism by 3.5, 3.6 and 4.4 folds, respectively	[188]
Titanium dioxide nanoparticles	Oral 2, 10 and 50 mg/kg to rats for 90 days	Affected lipid metabolism by lowering serum triglycerides, increasing lipid peroxidation marker malondialdehyde with no effect on serum total cholesterol, total cholesterol high density lipoprotein, and total cholesterol low density lipoprotein levels	[184]
	25, 50 and 100 µg/ml incubated with human bronchial epithelial cells (BEAS-2B)	Changed levels of sphingolipids, prenol lipids, fatty acyls and glycerophospholipids Enhanced cholesterol synthesis pathway	[185]
	10, 20, 50, 100 and 200 mg/kg BW orally to mice	Increased blood glucose levels starting from the dose of 50 mg/kg BW	[186]
	50 mg/kg BW orally to developing and adult mice	Hyperglycemia was more severe in developing mice than adult ones	[187]
	0.2 mmol/kg BW nasally administered to lactating mice from lactating days 1 to 12	Reduced lactogenesis and production of milk fat by 35.7%	[188]
	Oral 10 mg/kg BW	Raised levels of acetylornithine, sulfoxidized methionine and non-essential amino acids	[189]
Vanadium pentoxide nanoparticles	Human bronchial epithelial cells (BEAS-2B) were incubated with 0.01, 0.1 and 1 ppm for 24 h	Affected lipid metabolism by upregulating fatty acid oxidation, de novo fatty acids biosynthesis and downregulating glycosphingolipid metabolism Affected protein metabolism by upregulating amino sugar pathways	[190]
Cerium dioxide nanoparticles	Human bronchial epithelial cells (BEAS-2B) were exposed to 12.5 and 25 µg/ml for 6 h 0.5 and 2 mg/kg BW to mice for 4 h (acute) or 44 days (chronic)	Affected lipid metabolism increasing sphingosine-1-phosphate levels and its metabolites. In addition to promoting oxidation of fatty acids	[191]
Silica nanoparticles	Intratracheal administration of 1.5 mg/kg BW/week 3 mg/kg BW/week 6 mg/kg BW/week For 12 weeks	Reduced glutathione and elevated reactive oxygen species and malondialdehyde Disrupted amino acids metabolism	[192]
PLGA polymeric nanoparticles	Macrophages differentiated from human monocytic cell line (THP-1) were incubated with 100 µg/ml for 24 h	Poloxamer-coated nanoparticles increased levels of arginine, proline, and N4-acetylaminobutanal, while polyethylene glycol-coated ones showed less disturbance in amino acid metabolism	[193]

Despite NPs’ advantages and potential as antiviral agents, their cytotoxicity, oxidative stress, and metabolic disruption remain issues that need to be well addressed. *Lactobacillus rhamnosus* *GG* protected against hepatoxicity induced by TiO_2_NPs in four-week-old rats [194]. Selenium is a regulator of metabolic functions asides from its well-known antioxidant and immunostimulant actions [195, 196]. Its deficiency was linked to mortality in COVID-19 patients [197]. On the other hand, it successfully ameliorated hepatoxicity and oxidative stress induced by the exposure of rats to ZnO NPs [198]**.** Furthermore, beneficial supplementation of selenium was observed in hyperlipidemia management [199], hyperglycemia [200], and hyperphenylalaninemia [201]. Furthermore, Zhang et al. reported the promising management of abnormal lipid metabolism using selenium and magnesium co-supplementation. They attributed these findings to their potential to decrease serum and liver TC, TC-LDL and cholesterol endogenous synthesis via increasing lecithin cholesterol acyltransferase expression levels and decreasing 3-hydroxy-3-methylglutaryl coenzyme A (HMG-CoA) reductase activity [202]. Accordingly, such encouraging findings could open the perspectives to future utilization of selenium, magnesium, and *Lactobacillus rhamnosus GG* as supportive agents against metabolic perturbations caused by COVID-19.

### Future directions and conclusions

**Fig. 5:**
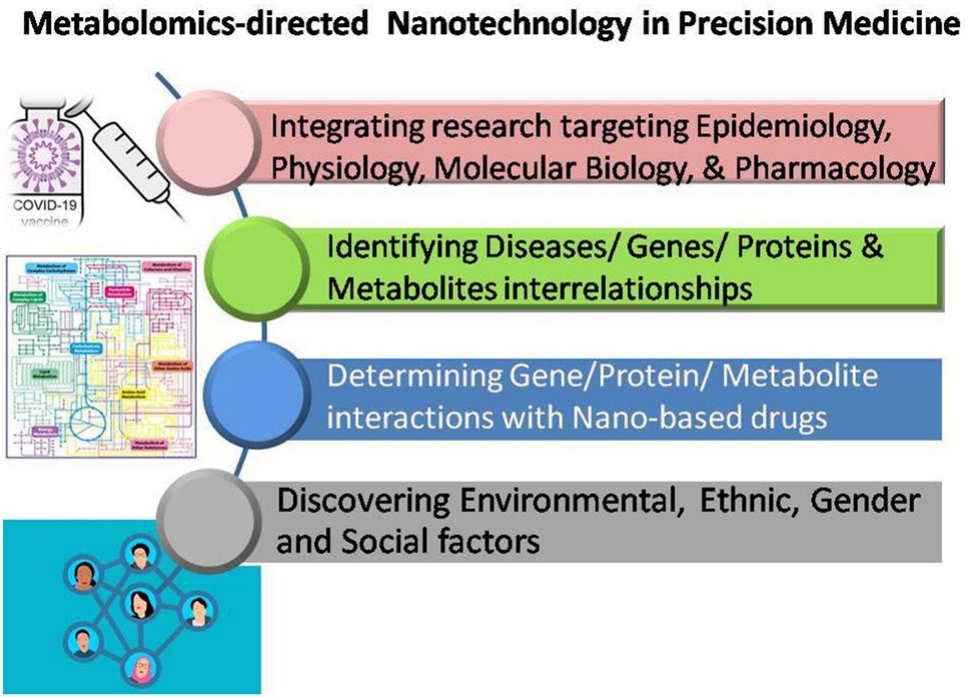
Future directions and challenges facing the science of metabolomics-directed nanotechnology aiming for the management of COVID-19 through precision medicine implementation

The necessity for implementing personalized (precision) medicine worldwide has been increasingly recognized. Hence, the integration of multidisciplinary studies is currently warranted to maximize the benefits of metabolomics-directed nanotechnology in COVID-19 management. Furthermore, better strategies should be considered to ensure its efficacy and likewise safety in different populations. Current challenges and upcoming future directions facing the fields of metabolomics and nanotechnology towards the development of precision medicine aiming for better management of COVID-19 are depicted in Fig. 5.

Briefly, metabolomics-directed nanotechnology is an emerging scientific field that needs to be evaluated from multiple perspectives including: epidemiology, physiology, molecular biology, bioethics, pharmacology and drug design. Indeed, several aspects need to be considered for better implementation of this newly developing technology. These include investigating the influence of heterogeneity in genetic/protein/metabolite profiling and their interrelationships with diseases. For instance, Oliveira et al. [203] determined several potential metabolic signatures distinguishing severe from non-severe COVID-19 cases to further identify the relationship between the metabolite profiling and the clinical and laboratory findings. This metabolomics analysis indicated that metabolites from porphyrin and purine pathways as well as glycerophospholipid and linoleic acid may exert prognostic and diagnostic merit in COVID-19.

Furthermore, metabolic signatures reprogramming in COVID-19 survivors and its association with long-term health consequences were previously examined [204]. Metabolic perturbations in organic acids, fatty acids, purines and amino acids were associated with dysregulated energy production, liver injury and inflammatory responses. These metabolic changes aided to identify potential biomarkers and putative therapeutic targets for COVID-19 management. In addition, the common key metabolomic and lipidomic changes were confirmed regardless of clinical symptoms’ heterogeneity in COVID-19 patients [205]. Likewise, several biomarkers such as fatty acids, lipids proteins and amino acids were strongly associated with susceptibility to COVID-19 severe cases [206].

Allover, these findings aided in developing an emerging field termed COVID-omics to address the role of proteomics and metabolomics in COVID diagnosis, prognosis, and management [207]. Survivors of COVID-19 manifest persistent dysregulation of metabolomic and proteomic systems even after six months following hospital discharge [27]. Severe COVID-19 is also associated with dysfunctional neutrophil metabolome as recently reported [208]. Furthermore, Bardanzellu and Fanos [209] suggested that machine learning, metabolomics and microbiomics, commonly known as the 3 M's will provide risk assessment and decision-making for COVID-19 diagnosis and therapy.

Using machine learning tools, Bennet et al. [210] conducted nasopharyngeal metabolomic to discriminate COVID-19 patients from Influenza A and respiratory syncytial virus; where levels of lysophosphatidylcholines were increased, whereas levels of β-hydroxybutyric acid, methionine sulfoxide, succinic acid, and carnosine were decreased in COVID-19 patients. Metabolomic investigation of plasma immune signatures revealed reduced levels of the acute phase and macrophage-induced proteins, concurrent with increased omega-3 fatty acid levels following three months of COVID-19 infection [211]. Similarly, several metabolic alterations were observed in COVID-19 patients’ serum samples compared to healthy subjects including dysregulated fatty acids, glycerophospholipids, and amino acids [212]. Lipidomic analysis of COVID-19 patients’ exosomes observed an enriched content of monosialodihexosyl ganglioside (GM3), which is postulated to play a role in COVID-19 pathogenesis [55]. Likewise, using salivary fingerprint was suggested for metabolomic profiling of COVID-19 patients [213]. Hence, the diversity in the suggested sample types and their metabolomic analysis with different time courses present a potential metabolomic matrix for experimental and clinical investigations.

Genetic/protein/metabolite interactions with nano-based drugs should also be addressed in the putative arsenals for combating COVID-19. For instance, nanoformulations of arachidonic acid and its metabolites might represent a safe and efficacious alternative for COVID-19 management [214]. Finally, identification of environmental, ethnic, gender and social factors affecting metabolomics of COVID-19 infection and management with nano-therapeutics is warranted for novel drug discoveries. Importantly, gender-based metabolic dysregulation was previously reported [215] including disrupted fatty acids metabolism in males and dysregulated glycerophosphocholines and carbohydrates metabolism in females. Interestingly, females exhibited a shorter duration of hospitalization compared to males, thus proposing a gender basis in COVID-19 prognosis and clinical outcome.

In conclusion, several in vitro*, *in vivo*,* and clinical studies revealed different key metabolites affected by COVID-19, thus confirming their biomarker role in the disease progression. Thus, exposure to nanoparticles is suggested as preventive or treatment regimen to modulate the profound metabolic disturbances associated with COVID-19. In this context, this review aims to present a holistic overview of the major metabolomic changes and the putative role of nanotechnology targeting SARS-COV-2 metabolomics dysregulation. Further predictive algorithms for biomarkers analysis and data interpretation are warranted to pave the way to COVID-19 personalized antiviral medicine and likewise pursue other viruses of the *Coronaviridae* family.

## Data Availability

Data sharing is not applicable to this article as no datasets were generated or analyzed during the current study.

## Abbreviations

AA: Arachidonic acid
ATP: Adenosine triphosphate
ACE2: Angiotensin-converting enzyme 2
Apo: Apolipoprotein
BW: Body weight
COVID-19: Coronavirus disease 2019
DHEAS: Dehydroepiandrosteronesulphate
GABA: Gamma amino butyric acid
HIF-1α: Hypoxia-inducible factor-1α
HMG-CoA: 3-Hydroxy-3-methylglutaryl coenzyme A
IFN: Interferon
IL: Interleukin
IP: Intraperitoneal
IV: Intravenous
HDL: High-density lipoprotein
LDL: Low-density lipoproteins
LPC: Lysophosphatidylcholine
MERS: Middle East respiratory syndrome
NPs: Nanoparticles
NAD+: Nicotinamide adenine dinucleotide
PC: Phosphatidylcholine
PEDV: Porcine epidemic diarrhea virus
PUFAs: Polyunsaturated fatty acids
ROS: Reactive oxygen species
SARS-CoV-2: Severe acute respiratory syndrome coronavirus 2
S1P: Sphingosine-1-phosphate
TCA: Tricarboxylic acid
TG: Triglycerides
TC: Total cholesterol
TLR: Toll-like receptor
TNF-α: Tumor necrosis factor-alpha
VLDL: Very-low density lipoprotein

## References

[1] Sarkar J, Das S, Aich S, Bhattacharyya P, Acharya K (2022). Antiviral potential of nanoparticles for the treatment of coronavirus infections. J Trace Elem Med Biol.

[2] Wang L, Wang Y, Ye D, Liu Q (2020). Review of the 2019 novel coronavirus (SARS-CoV-2) based on current evidence. Int J Antimicrob Agents.

[3] Chen N, Zhou M, Dong X, Qu J, Gong F, Han Y (2020). Epidemiological and clinical characteristics of 99 cases of 2019 novel coronavirus pneumonia in Wuhan, China: a descriptive study. Lancet.

[4] Laurencin CT, McClinton A (2020). The COVID-19 pandemic: a call to action to identify and address racial and ethnic disparities. J Racial Ethn Health Disparities.

[5] Hasan MR, Suleiman M, Pérez-López A (2021). Metabolomics in the diagnosis and prognosis of COVID-19. Front Genet.

[6] Nicholson JK (2021). Molecular phenomic approaches to deconvolving the systemic effects of SARS-CoV-2 infection and post-acute COVID-19 syndrome. Phenomics.

[7] Ward RA , Aghaeepour N, Bhattacharyya RP, Clish CB, Gaudillière B, Hacohen N et al. Harnessing the potential of multiomics studies for precision medicine in infectious disease. open forum infectious diseases. 2021; 8(11):ofab483. 10.1093/ofid/ofab483 PMC8598922 34805429

[8] Bruzzone C, Conde R, Embade N, Mato JM, Millet O (2023). Metabolomics as a powerful tool for diagnostic, pronostic and drug intervention analysis in COVID-19. Front Mol Biosci.

[9] Clish CB (2015). Metabolomics: an emerging but powerful tool for precision medicine. Cold Spring Harb Mol Case Stud.

[10] Hu J, Peng P, Cao X, Wu K, Chen J, Wang K (2022). Increased immune escape of the new SARS-CoV-2 variant of concern Omicron. Cell Mol Immunol.

[11] Binayke A, Zaheer A, Dandotiya J, Gupta SK, Mani S, Tripathy MR, et al. Proinflammatory innate cytokines and distinct metabolomic signatures shape the T cell response in active COVID-19. Vaccines (Basel). 2022;10(10). 10.3390/vaccines10101762 PMC9609972 36298628

[12] Nasrollahzadeh M, Sajjadi M, Soufi GJ, Iravani S, Varma RS. Nanomaterials and nanotechnology-associated innovations against viral infections with a focus on coronaviruses. Nanomaterials (Basel). 2020;10(6). 10.3390/nano10061072 PMC7352498 32486364

[13] Verbeke R, Lentacker I, De Smedt SC, Dewitte H (2021). The dawn of mRNA vaccines: the COVID-19 case. J Control Release.

[14] Gomes A, Sengupta J, Datta P, Ghosh S, Gomes A (2016). Physiological interactions of nanoparticles in energy metabolism, immune function and their biosafety: a review. J Nanosci Nanotechnol.

[15] Gülpınar Ö, Güçlü AG (2013). How to write a review article?. Turk J Urol.

[16] Lorizate M, Kräusslich HG (2011). Role of lipids in virus replication. Cold Spring Harb Perspect Biol.

[17] Sorokin AV, Karathanasis SK, Yang ZH, Freeman L, Kotani K, Remaley AT (2020). COVID-19-associated dyslipidemia: implications for mechanism of impaired resolution and novel therapeutic approaches. FASEB J.

[18] Shen B, Yi X, Sun Y, Bi X, Du J, Zhang C (2020). Proteomic and metabolomic characterization of COVID-19 patient sera. Cell.

[19] Caterino M, Costanzo M, Fedele R, Cevenini A, Gelzo M, Di Minno A, et al. The serum metabolome of moderate and severe COVID-19 patients reflects possible liver alterations involving carbon and nitrogen metabolism. Int J Mol Sci. 2021;22(17). 10.3390/ijms22179548 PMC8431319 34502454

[20] Tomo S, Banerjee M, Karli S, Purohit P, Mitra P, Sharma P (2022). Assessment of DHEAS, cortisol, and DHEAS/cortisol ratio in patients with COVID-19: a pilot study. Hormones (Athens).

[21] Jin JM, Bai P, He W, Wu F, Liu XF, Han DM (2020). Gender differences in patients with COVID-19: focus on severity and mortality. Front Public Health.

[22] Tomo S, Banerjee M, Sharma P, Garg M (2021). Does dehydroepiandrosterone sulfate have a role in COVID-19 prognosis and treatment?. Endocr Regul.

[23] Auci D, Kaler L, Subramanian S, Huang Y, Frincke J, Reading C (2007). A new orally bioavailable synthetic androstene inhibits collagen-induced arthritis in the mouse: androstene hormones as regulators of regulatory T cells. Ann N Y Acad Sci.

[24] Schmelter F, Föh B, Mallagaray A, Rahmöller J, Ehlers M, Lehrian S (2021). Metabolic and lipidomic markers differentiate COVID-19 from non-hospitalized and other intensive care patients. Front Mol Biosci.

[25] Bruzzone C, Bizkarguenaga M, Gil-Redondo R, Diercks T, Arana E, García de Vicuña A, et al. SARS-CoV-2 infection dysregulates the metabolomic and lipidomic profiles of serum. iScience. 2020;23(10):101645. 10.1016/j.isci.2020.101645 PMC7534591 33043283

[26] Ghini V, Meoni G, Pelagatti L, Celli T, Veneziani F, Petrucci F (2022). Profiling metabolites and lipoproteins in COMETA, an Italian cohort of COVID-19 patients. PLoS Pathog.

[27] Li H, Li X, Wu Q, Wang X, Qin Z, Wang Y (2022). Plasma proteomic and metabolomic characterization of COVID-19 survivors 6 months after discharge. Cell Death Dis.

[28] Yu X, Xu X, Wu T, Huang W, Xu C, Xie W (2022). APOA1 level is negatively correlated with the severity of COVID-19. Int J Gen Med.

[29] Ciccarelli M, Merciai F, Carrizzo A, Sommella E, Di Pietro P, Caponigro V (2022). Untargeted lipidomics reveals specific lipid profiles in COVID-19 patients with different severity from Campania region (Italy). J Pharm Biomed Anal.

[30] Bizkarguenaga M, Bruzzone C, Gil-Redondo R, SanJuan I, Martin-Ruiz I, Barriales D (2022). Uneven metabolic and lipidomic profiles in recovered COVID-19 patients as investigated by plasma NMR metabolomics. NMR Biomed.

[31] Richardson TG, Fang S, Mitchell RE, Holmes MV, Davey SG (2021). Evaluating the effects of cardiometabolic exposures on circulating proteins which may contribute to severe SARS-CoV-2. EBioMedicine.

[32] Wu Q, Zhou L, Sun X, Yan Z, Hu C, Wu J (2017). Altered lipid metabolism in recovered SARS patients twelve years after infection. Sci Rep.

[33] Valdés A, Moreno LO, Rello SR, Orduña A, Bernardo D, Cifuentes A (2022). Metabolomics study of COVID-19 patients in four different clinical stages. Sci Rep.

[34] Bennett M, Gilroy DW. Lipid mediators in inflammation. Microbiol Spectr. 2016;4(6). 10.1128/microbiolspec.MCHD-0035-2016 27837747

[35] Masoodi M, Peschka M, Schmiedel S, Haddad M, Frye M, Maas C (2022). Disturbed lipid and amino acid metabolisms in COVID-19 patients. J Mol Med (Berl).

[36] Das UN (2020). Can bioactive lipids inactivate coronavirus (COVID-19)?. Arch Med Res.

[37] Casari I, Manfredi M, Metharom P, Falasca M (2021). Dissecting lipid metabolism alterations in SARS-CoV-2. Prog Lipid Res.

[38] Castañé H, Iftimie S, Baiges-Gaya G, Rodríguez-Tomàs E, Jiménez-Franco A, López-Azcona AF (2022). Machine learning and semi-targeted lipidomics identify distinct serum lipid signatures in hospitalized COVID-19-positive and COVID-19-negative patients. Metabolism.

[39] Karu N, Kindt A, Lamont L, van Gammeren AJ, Ermens AAM, Harms AC, et al. Plasma oxylipins and their precursors are strongly associated with COVID-19 severity and with immune response markers. Metabolites. 2022;12(7). 10.3390/metabo12070619 PMC9319897 35888743

[40] Palmas F, Clarke J, Colas RA, Gomez EA, Keogh A, Boylan M (2021). Dysregulated plasma lipid mediator profiles in critically ill COVID-19 patients. PLoS ONE.

[41] Pérez MM, Pimentel VE, Fuzo CA, da Silva-Neto PV, Toro DM, Fraga-Silva TFC, et al. Acetylcholine, fatty acids, and lipid. 2022mediators are linked to COVID-19 severity. J Immunol. 2022;209(2):250–61. 10.4049/jimmunol.2200079 35768148

[42] Rhen T, Cidlowski JA (2005). Antiinflammatory action of glucocorticoids—new mechanisms for old drugs. N Engl J Med.

[43] Reis MB, Rodrigues FL, Lautherbach N, Kanashiro A, Sorgi CA, Meirelles AFG (2020). Interleukin-1 receptor-induced PGE_2_ production controls acetylcholine-mediated cardiac dysfunction and mortality during scorpion envenomation. Nat Commun.

[44] Heffernan KS, Ranadive SM, Jae SY (2020). Exercise as medicine for COVID-19: On PPAR with emerging pharmacotherapy. Med Hypotheses.

[45] Wang Q, He Y, Shen Y, Zhang Q, Chen D, Zuo C (2014). Vitamin D inhibits COX-2 expression and inflammatory response by targeting thioesterase superfamily member 4. J Biol Chem.

[46] Leghmar K, Cenac N, Rolland M, Martin H, Rauwel B, Bertrand-Michel J (2015). Cytomegalovirus infection triggers the secretion of the PPARγ agonists 15-hydroxyeicosatetraenoic acid (15-HETE) and 13-hydroxyoctadecadienoic acid (13-HODE) in human cytotrophoblasts and placental cultures. PLoS ONE.

[47] Yeung J, Hawley M, Holinstat M (2017). The expansive role of oxylipins on platelet biology. J Mol Med (Berl).

[48] Thomas T, Stefanoni D, Reisz JA, Nemkov T, Bertolone L, Francis RO, et al. COVID-19 infection alters kynurenine and fatty acid metabolism, correlating with IL-6 levels and renal status. JCI Insight. 2020;5(14). 10.1172/jci.insight.140327 PMC7453907 32559180

[49] Archambault AS, Zaid Y, Rakotoarivelo V, Turcotte C, Doré É, Dubuc I (2021). High levels of eicosanoids and docosanoids in the lungs of intubated COVID-19 patients. FASEB J.

[50] Iftimie S, García-Heredia A, Pujol I, Ballester F, Fort-Gallifa I, Simó JM (2016). Preliminary study on serum paraoxonase-1 status and chemokine (C-C motif) ligand 2 in hospitalized elderly patients with catheter-associated asymptomatic bacteriuria. Eur J Clin Microbiol Infect Dis.

[51] Mai M, Tönjes A, Kovacs P, Stumvoll M, Fiedler GM, Leichtle AB (2013). Serum levels of acylcarnitines are altered in prediabetic conditions. PLoS ONE.

[52] Ayres JS (2020). A metabolic handbook for the COVID-19 pandemic. Nat Metab.

[53] Barberis E, Timo S, Amede E, Vanella VV, Puricelli C, Cappellano G, et al. Large-scale plasma analysis revealed new mechanisms and molecules associated with the host response to SARS-CoV-2. Int J Mol Sci. 2020;21(22). 10.3390/ijms21228623 PMC7696386 33207699

[54] Otsubo C, Bharathi S, Uppala R, Ilkayeva OR, Wang D, McHugh K (2015). Long-chain acylcarnitines reduce lung function by inhibiting pulmonary surfactant. J Biol Chem.

[55] Song JW, Lam SM, Fan X, Cao WJ, Wang SY, Tian H (2020). Omics-driven systems interrogation of metabolic dysregulation in COVID-19 pathogenesis. Cell Metab.

[56] Deguchi H, Banerjee Y, Trauger S, Siuzdak G, Kalisiak E, Fernández JA (2015). Acylcarnitines are anticoagulants that inhibit factor Xa and are reduced in venous thrombosis, based on metabolomics data. Blood.

[57] Kong F, Saif LJ, Wang Q (2021). Roles of bile acids in enteric virus replication. Anim Dis.

[58] Herold BC, Kirkpatrick R, Marcellino D, Travelstead A, Pilipenko V, Krasa H (1999). Bile salts: natural detergents for the prevention of sexually transmitted diseases. Antimicrob Agents Chemother.

[59] Luo L, Han W, Du J, Yang X, Duan M, Xu C, et al. Chenodeoxycholic acid from bile inhibits influenza a virus replication via blocking nuclear export of viral ribonucleoprotein complexes. Molecules. 2018;23(12). 10.3390/molecules23123315 PMC6321071 30558117

[60] Reese VC, Oropeza CE, McLachlan A (2013). Independent activation of hepatitis B virus biosynthesis by retinoids, peroxisome proliferators, and bile acids. J Virol.

[61] Giron LB, Dweep H, Yin X, Wang H, Damra M, Goldman AR (2021). Plasma markers of disrupted gut permeability in severe COVID-19 patients. Front Immunol.

[62] Sanchez-Lopez E, Zhong Z, Stubelius A, Sweeney SR, Booshehri LM, Antonucci L (2019). Choline uptake and metabolism modulate macrophage IL-1β and IL-18 production. Cell Metab.

[63] Savelli G, Bonacina M, Rizzo A, Zaniboni A. Activated macrophages are the main inflammatory cell in COVID-19 interstitial pneumonia infiltrates. Is it possible to show their metabolic activity and thus the grade of inflammatory burden with (18)F-fluorocholine PET/CT? Med Hypotheses. 2020;144:109885. 10.1016/j.mehy.2020.109885 PMC7252431 32540605

[64] Fraser DD, Slessarev M, Martin CM, Daley M, Patel MA, Miller MR (2020). Metabolomics profiling of critically ill coronavirus disease 2019 patients: identification of diagnostic and prognostic biomarkers. Crit Care Explor.

[65] Delafiori J, Navarro LC, Siciliano RF, de Melo GC, Busanello ENB, Nicolau JC (2021). Covid-19 automated diagnosis and risk assessment through metabolomics and machine learning. Anal Chem.

[66] Wu D, Shu T, Yang X, Song JX, Zhang M, Yao C (2020). Plasma metabolomic and lipidomic alterations associated with COVID-19. Natl Sci Rev.

[67] Lam SM, Zhang C, Wang Z, Ni Z, Zhang S, Yang S (2021). A multi-omics investigation of the composition and function of extracellular vesicles along the temporal trajectory of COVID-19. Nat Metab.

[68] Cai Y, Kim DJ, Takahashi T, Broadhurst DI, Yan H, Ma S, et al. Kynurenic acid may underlie sex-specific immune responses to COVID-19. Sci Signal. 2021;14(690). 10.1126/scisignal.abf8483 PMC8432948 34230210

[69] Hao Y, Zhang Z, Feng G, Chen M, Wan Q, Lin J, et al. Distinct lipid metabolic dysregulation in asymptomatic COVID-19. iScience. 2021;24(9):102974. 10.1016/j.isci.2021.102974 PMC8356725 34396083

[70] Žarković N, Orehovec B, Baršić B, Tarle M, Kmet M, Lukšić I, et al. Lipidomics revealed plasma phospholipid profile differences between deceased and recovered COVID-19 patients. Biomolecules. 2022;12(10). 10.3390/biom12101488 PMC9599609 36291697

[71] Wei J, Liu X, Xiao W, Lu J, Guan L, Fang Z (2023). Phospholipid remodeling and its derivatives are associated with COVID-19 severity. J Allergy Clin Immunol.

[72] Hannun YA, Obeid LM (2018). Sphingolipids and their metabolism in physiology and disease. Nat Rev Mol Cell Biol.

[73] Barnawi J, Tran H, Jersmann H, Pitson S, Roscioli E, Hodge G (2015). Potential link between the sphingosine-1-phosphate (S1P) system and defective alveolar macrophage phagocytic function in chronic obstructive pulmonary disease (COPD). PLoS ONE.

[74] Gomez-Gomez A, Rodríguez-Morató J, Haro N, Marín-Corral J, Masclans JR, Pozo OJ (2022). Untargeted detection of the carbonyl metabolome by chemical derivatization and liquid chromatography-tandem mass spectrometry in precursor ion scan mode: elucidation of COVID-19 severity biomarkers. Anal Chim Acta.

[75] Khodadoust MM (2021). Inferring a causal relationship between ceramide levels and COVID-19 respiratory distress. Sci Rep.

[76] Marfia G, Navone S, Guarnaccia L, Campanella R, Mondoni M, Locatelli M (2021). Decreased serum level of sphingosine-1-phosphate: a novel predictor of clinical severity in COVID-19. EMBO Mol Med.

[77] Naz F, Arish M (2020). Battling COVID-19 pandemic: sphingosine-1-phosphate analogs as an adjunctive therapy?. Front Immunol.

[78] Rao MJ, Tahir Ul Qamar M, Wang D, Ali Q, Ma L, Han S, et al. A high-throughput lipidomics and transcriptomic approach reveals novel compounds from sugarcane linked with promising therapeutic potential against COVID-19. Front Nutr. 2022;9:988249. 10.3389/fnut.2022.988249 PMC9480494 36118771

[79] Tahir Ul Qamar M, Alqahtani SM, Alamri MA, Chen LL. Structural basis of SARS-CoV-2 3CL^pro^and anti-COVID-19 drug discovery from medicinal plants. J Pharm Anal. 2020;10(4):313–9. 10.1016/j.jpha.2020.03.009 PMC7156227 32296570

[80] Ansone L, Briviba M, Silamikelis I, Terentjeva A, Perkons I, Birzniece L (2021). Amino acid metabolism is significantly altered at the time of admission in hospital for severe COVID-19 patients: findings from longitudinal targeted metabolomics analysis. Microbiol Spectr.

[81] Danlos FX, Grajeda-Iglesias C, Durand S, Sauvat A, Roumier M, Cantin D (2021). Metabolomic analyses of COVID-19 patients unravel stage-dependent and prognostic biomarkers. Cell Death Dis.

[82] Xiao N, Nie M, Pang H, Wang B, Hu J, Meng X (2021). Integrated cytokine and metabolite analysis reveals immunometabolic reprogramming in COVID-19 patients with therapeutic implications. Nat Commun.

[83] Turski WA, Wnorowski A, Turski GN, Turski CA, Turski L (2020). AhR and IDO1 in pathogenesis of Covid-19 and the "Systemic AhR Activation Syndrome:" a translational review and therapeutic perspectives. Restor Neurol Neurosci.

[84] Bi X, Liu W, Ding X, Liang S, Zheng Y, Zhu X (2022). Proteomic and metabolomic profiling of urine uncovers immune responses in patients with COVID-19. Cell Rep.

[85] Morrison EJ, Champagne DP, Dzieciatkowska M, Nemkov T, Zimring JC, Hansen KC, et al. Parabiosis incompletely reverses aging-induced metabolic changes and oxidant stress in mouse red blood cells. Nutrients. 2019;11(6). 10.3390/nu11061337 PMC6627295 31207887

[86] Minhas PS, Liu L, Moon PK, Joshi AU, Dove C, Mhatre S (2019). Macrophage de novo NAD^+^ synthesis specifies immune function in aging and inflammation. Nat Immunol.

[87] Lee GK, Park HJ, Macleod M, Chandler P, Munn DH, Mellor AL (2002). Tryptophan deprivation sensitizes activated T cells to apoptosis prior to cell division. Immunology.

[88] Van Gool F, Gallí M, Gueydan C, Kruys V, Prevot PP, Bedalov A (2009). Intracellular NAD levels regulate tumor necrosis factor protein synthesis in a sirtuin-dependent manner. Nat Med.

[89] Greene LI, Bruno TC, Christenson JL, D'Alessandro A, Culp-Hill R, Torkko K (2019). A role for tryptophan-2,3-dioxygenase in CD8 T-cell suppression and evidence of tryptophan catabolism in breast cancer patient plasma. Mol Cancer Res.

[90] Sorgdrager FJH, Naudé PJW, Kema IP, Nollen EA, Deyn PP. Tryptophan metabolism in inflammaging: from biomarker to therapeutic target. Front Immunol. 2019;10:2565. 10.3389/fimmu.2019.02565 PMC6833926 31736978

[91] Jia H, Liu C, Li D, Huang Q, Liu D, Zhang Y, et al. Metabolomic analyses reveal new stage-specific features of COVID-19. Eur Respir J. 2022;59(2). 10.1183/13993003.00284-2021 PMC8311281 34289974

[92] Li T, Ning N, Li B, Luo D, Qin E, Yu W (2021). Longitudinal metabolomics reveals ornithine cycle dysregulation correlates with inflammation and coagulation in COVID-19 severe patients. Front Microbiol.

[93] Bourgin M, Derosa L, Silva CAC, Goubet AG, Dubuisson A, Danlos FX (2021). Circulating acetylated polyamines correlate with Covid-19 severity in cancer patients. Aging (Albany NY).

[94] Reyes AA, Karl IE, Klahr S (1994). Role of arginine in health and in renal disease. Am J Physiol.

[95] Páez-Franco JC, Torres-Ruiz J, Sosa-Hernández VA, Cervantes-Díaz R, Romero-Ramírez S, Pérez-Fragoso A (2021). Metabolomics analysis reveals a modified amino acid metabolism that correlates with altered oxygen homeostasis in COVID-19 patients. Sci Rep.

[96] Fan TW, Lane AN, Higashi RM, Farag MA, Gao H, Bousamra M (2009). Altered regulation of metabolic pathways in human lung cancer discerned by ^13^C stable isotope-resolved metabolomics (SIRM). Mol Cancer.

[97] Correia BSB, Ferreira VG, Piagge P, Almeida MB, Assunção NA, Raimundo JRS (2022). (1)H qNMR-based metabolomics discrimination of covid-19 severity. J Proteome Res.

[98] Luporini RL, Pott-Junior H, Di Medeiros Leal MCB, Castro A, Ferreira AG, Cominetti MR (2021). Phenylalanine and COVID-19: Tracking disease severity markers. Int Immunopharmacol.

[99] Gostner JM, Becker K, Kurz K, Fuchs D (2015). Disturbed amino acid metabolism in HIV: association with neuropsychiatric symptoms. Front Psychiatry.

[100] Sadeghi M, Lahdou I, Daniel V, Schnitzler P, Fusch G, Schefold JC (2012). Strong association of phenylalanine and tryptophan metabolites with activated cytomegalovirus infection in kidney transplant recipients. Hum Immunol.

[101] Geisler S, Gostner JM, Becker K, Ueberall F, Fuchs D (2013). Immune activation and inflammation increase the plasma phenylalanine-to-tyrosine ratio. Pteridines.

[102] Cavalli G, Justice JN, Boyle KE, D'Alessandro A, Eisenmesser EZ, Herrera JJ (2017). Interleukin 37 reverses the metabolic cost of inflammation, increases oxidative respiration, and improves exercise tolerance. Proc Natl Acad Sci USA.

[103] Bertolone L, Roy MK, Hay AM, Morrison EJ, Stefanoni D, Fu X (2020). Impact of taurine on red blood cell metabolism and implications for blood storage. Transfusion.

[104] Klempin F, Mosienko V, Matthes S, Villela DC, Todiras M, Penninger JM (2018). Depletion of angiotensin-converting enzyme 2 reduces brain serotonin and impairs the running-induced neurogenic response. Cell Mol Life Sci.

[105] Nataf S (2020). An alteration of the dopamine synthetic pathway is possibly involved in the pathophysiology of COVID-19. J Med Virol.

[106] Attademo L, Bernardini F (2021). Are dopamine and serotonin involved in COVID-19 pathophysiology?. Eur J Psychiatry.

[107] Zheng Y, Zhang Y, Chi H, Chen S, Peng M, Luo L (2020). The hemocyte counts as a potential biomarker for predicting disease progression in COVID-19: a retrospective study. Clin Chem Lab Med.

[108] Hamed MGM, Hagag RS (2020). The possible immunoregulatory and anti-inflammatory effects of selective serotonin reuptake inhibitors in coronavirus disease patients. Med Hypotheses.

[109] Nicolau GY, Haus E, Lakatua D, Sackett-Lundeen L, Bogdan C, Plingă L (1985). Differences in the circadian rhythm parameters of urinary free epinephrine, norepinephrine and dopamine between children and elderly subjects. Endocrinologie.

[110] Derakhshan M, Ansarian HR, Ghomshei M (2020). Possible effect of epinephrine in minimizing COVID-19 severity: a review. J Int Med Res.

[111] Dimitrov S, Benedict C, Heutling D, Westermann J, Born J, Lange T (2009). Cortisol and epinephrine control opposing circadian rhythms in T cell subsets. Blood.

[112] Flierl MA, Rittirsch D, Nadeau BA, Chen AJ, Sarma JV, Zetoune FS (2007). Phagocyte-derived catecholamines enhance acute inflammatory injury. Nature.

[113] Staedtke V, Bai RY, Kim K, Darvas M, Davila ML, Riggins GJ (2018). Disruption of a self-amplifying catecholamine loop reduces cytokine release syndrome. Nature.

[114] Luo P, Liu D, Li J (2021). Epinephrine use in COVID-19: friend or foe?. Eur J Hosp Pharm.

[115] Shi J, Fan J, Su Q, Yang Z (2019). Cytokines and abnormal glucose and lipid metabolism. Front Endocrinol (Lausanne).

[116] Zhu L, She ZG, Cheng X, Qin JJ, Zhang XJ, Cai J (2020). Association of blood glucose control and outcomes in patients with COVID-19 and pre-existing type 2 diabetes. Cell Metab.

[117] Smith SM, Boppana A, Traupman JA, Unson E, Maddock DA, Chao K (2021). Impaired glucose metabolism in patients with diabetes, prediabetes, and obesity is associated with severe COVID-19. J Med Virol.

[118] Codo AC, Davanzo GG, Monteiro LdB, de Souza GF, Muraro SP, Virgilio-da-Silva JV, et al. Elevated glucose levels favor SARS-CoV-2 infection and monocyte response through a HIF-1α/glycolysis-dependent axis. Cell metabolism. 2020;32(3):498–9. 10.1016/j.cmet.2020.07.015 PMC7462530 32877692

[119] Bojkova D, Klann K, Koch B, Widera M, Krause D, Ciesek S (2020). Proteomics of SARS-CoV-2-infected host cells reveals therapy targets. Nature.

[120] Li Z, Liu G, Wang L, Liang Y, Zhou Q, Wu F (2020). From the insight of glucose metabolism disorder: oxygen therapy and blood glucose monitoring are crucial for quarantined COVID-19 patients. Ecotoxicol Environ Saf.

[121] Martínez-Reyes I, Chandel NS (2020). Mitochondrial TCA cycle metabolites control physiology and disease. Nat Commun.

[122] Zhang K, Liu X, Shen J, Li Z, Sang Y, Wu X (2020). Clinically applicable AI system for accurate diagnosis, quantitative measurements, and prognosis of COVID-19 pneumonia using computed tomography. Cell.

[123] Jang K-J, Jeong S, Kang DY, Sp N, Yang YM, Kim D-E (2020). A high ATP concentration enhances the cooperative translocation of the SARS coronavirus helicase nsP13 in the unwinding of duplex RNA. Sci Rep.

[124] Lewis HM, Liu Y, Frampas CF, Longman K, Spick M, Stewart A, et al. Metabolomics markers of COVID-19 are dependent on collection wave. Metabolites. 2022;12(8). 10.3390/metabo12080713 PMC9415837 36005585

[125] Liu R, Luo C, Pang Z, Zhang J, Ruan S, Wu M (2023). Advances of nanoparticles as drug delivery systems for disease diagnosis and treatment. Chin Chem Lett.

[126] Ge X, Cao Z, Chu L. The antioxidant effect of the metal and metal-oxide nanoparticles. Antioxidants (Basel). 2022;11(4). 10.3390/antiox11040791 PMC9030860 35453476

[127] Nishimoto-Sauceda D, Romero-Robles LE, Antunes-Ricardo M (2022). Biopolymer nanoparticles: a strategy to enhance stability, bioavailability, and biological effects of phenolic compounds as functional ingredients. J Sci Food Agric.

[128] Musielak E, Feliczak-Guzik A, Nowak I. Synthesis and potential applications of lipid nanoparticles in medicine. Materials (Basel). 2022;15(2). 10.3390/ma15020682 PMC8780297 35057398

[129] Botequim D, Maia J, Lino MM, Lopes LM, Simões PN, Ilharco LM (2012). Nanoparticles and surfaces presenting antifungal, antibacterial and antiviral properties. Langmuir.

[130] Yah CS, Simate GS (2015). Nanoparticles as potential new generation broad spectrum antimicrobial agents. Daru.

[131] Antoine TE, Hadigal SR, Yakoub AM, Mishra YK, Bhattacharya P, Haddad C (2016). Intravaginal zinc oxide tetrapod nanoparticles as novel immunoprotective agents against genital herpes. J Immunol.

[132] Khurana A, Tekula S, Saifi MA, Venkatesh P, Godugu C (2019). Therapeutic applications of selenium nanoparticles. Biomed Pharmacother.

[133] Chen L, Liang J (2020). An overview of functional nanoparticles as novel emerging antiviral therapeutic agents. Mater Sci Eng C Mater Biol Appl.

[134] Alavi M, Kamarasu P, McClements DJ, Moore MD (2022). Metal and metal oxide-based antiviral nanoparticles: properties, mechanisms of action, and applications. Adv Colloid Interface Sci.

[135] Chakravarty M, Vora A (2021). Nanotechnology-based antiviral therapeutics. Drug Deliv Transl Res.

[136] Szunerits S, Barras A, Khanal M, Pagneux Q, Boukherroub R (2015). Nanostructures for the inhibition of viral infections. Molecules.

[137] Ianevski A, Yao R, Fenstad MH, Biza S, Zusinaite E, Reisberg T, et al. Potential antiviral options against SARS-CoV-2 infection. Viruses. 2020;12(6). 10.3390/v12060642 PMC7354438 32545799

[138] Cojocaru FD, Botezat D, Gardikiotis I, Uritu CM, Dodi G, Trandafir L, et al. Nanomaterials designed for antiviral drug delivery transport across biological barriers. Pharmaceutics. 2020;12(2). 10.3390/pharmaceutics12020171 PMC7076512 32085535

[139] Bawage SS, Tiwari PM, Singh A, Dixit S, Pillai SR, Dennis VA (2016). Gold nanorods inhibit respiratory syncytial virus by stimulating the innate immune response. Nanomedicine.

[140] Halder A, Das S, Ojha D, Chattopadhyay D, Mukherjee A (2018). Highly monodispersed gold nanoparticles synthesis and inhibition of herpes simplex virus infections. Mater Sci Eng C Mater Biol Appl.

[141] Gaikwad S, Ingle A, Gade A, Rai M, Falanga A, Incoronato N (2013). Antiviral activity of mycosynthesized silver nanoparticles against herpes simplex virus and human parainfluenza virus type 3. Int J Nanomed.

[142] Hu RL, Li SR, Kong FJ, Hou RJ, Guan XL, Guo F (2014). Inhibition effect of silver nanoparticles on herpes simplex virus 2. Genet Mol Res.

[143] Haggag EG, Elshamy AM, Rabeh MA, Gabr NM, Salem M, Youssif KA (2019). Antiviral potential of green synthesized silver nanoparticles of *Lampranthus coccineus* and *Malephora lutea*. Int J Nanomed.

[144] Ratan ZA, Mashrur FR, Chhoan AP, Shahriar SM, Haidere MF, Runa NJ, et al. Silver nanoparticles as potential antiviral agents. pharmaceutics. 2021;13(12). 10.3390/pharmaceutics13122034 PMC8705988 34959320

[145] Farouk F, Shebl RI (2018). Comparing surface chemical modifications of zinc oxide nanoparticles for modulating their antiviral activity against herpes simplex virus type-1. Int J Nanopart Nanotechnol.

[146] Ghaffari H, Tavakoli A, Moradi A, Tabarraei A, Bokharaei-Salim F, Zahmatkeshan M (2019). Inhibition of H1N1 influenza virus infection by zinc oxide nanoparticles: another emerging application of nanomedicine. J Biomed Sci.

[147] Mazurkova NA, Spitsyna YE, Shikina NV, Ismagilov ZR, Zagrebel’nyi SN, Ryabchikova EI. Interaction of titanium dioxide nanoparticles with influenza virus. Nanotechnol Russia. 2010;5(5):417–20.

[148] Akhtar S, Shahzad K, Mushtaq S, Ali I, Rafe MH, Fazal-ul-Karim SM (2019). Antibacterial and antiviral potential of colloidal titanium dioxide (TiO_2_) nanoparticles suitable for biological applications. Mater Res Express.

[149] Li Y, Lin Z, Gong G, Guo M, Xu T, Wang C (2019). Inhibition of H1N1 influenza virus-induced apoptosis by selenium nanoparticles functionalized with arbidol through ROS-mediated signaling pathways. J Mater Chem B.

[150] Wang C, Chen H, Chen D, Zhao M, Lin Z, Guo M (2020). The inhibition of H1N1 influenza virus-induced apoptosis by surface decoration of selenium nanoparticles with β-thujaplicin through reactive oxygen species-mediated AKT and p53 signaling pathways. ACS Omega.

[151] Gurunathan S, Qasim M, Choi Y, Do JT, Park C, Hong K, et al. Antiviral potential of nanoparticles-can nanoparticles fight against coronaviruses? Nanomaterials (Basel). 2020;10(9). 10.3390/nano10091645 PMC7557932 32825737

[152] Reina G, Peng S, Jacquemin L, Andrade AF, Bianco A (2020). Hard nanomaterials in time of viral pandemics. ACS Nano.

[153] Hamdi M, Abdel-Bar HM, Elmowafy E, El-Khouly A, Mansour M, Awad GAS (2021). Investigating the internalization and COVID-19 antiviral computational analysis of optimized nanoscale zinc oxide. ACS Omega.

[154] Szymańska E, Orłowski P, Winnicka K, Tomaszewska E, Bąska P, Celichowski G, et al. Multifunctional tannic acid/silver nanoparticle-based mucoadhesive hydrogel for improved local treatment of HSV infection: in vitro and in vivo studies. Int J Mol Sci. 2018;19(2). 10.3390/ijms19020387 PMC5855609 29382085

[155] Wang Y, Canady TD, Zhou Z, Tang Y, Price DN, Bear DG (2011). Cationic phenylene ethynylene polymers and oligomers exhibit efficient antiviral activity. ACS Appl Mater Interfaces.

[156] Bimbo LM, Denisova OV, Mäkilä E, Kaasalainen M, De Brabander JK, Hirvonen J (2013). Inhibition of influenza A virus infection in vitro by saliphenylhalamide-loaded porous silicon nanoparticles. ACS Nano.

[157] Ahmed SR, Nagy É, Neethirajan S (2017). Self-assembled star-shaped chiroplasmonic gold nanoparticles for an ultrasensitive chiro-immunosensor for viruses. RSC Adv.

[158] Du T, Liang J, Dong N, Lu J, Fu Y, Fang L (2018). Glutathione-capped Ag_2_S nanoclusters inhibit coronavirus proliferation through blockage of viral RNA synthesis and budding. ACS Appl Mater Interfaces.

[159] Heinrich MA, Martina B, Prakash J (2020). Nanomedicine strategies to target coronavirus. Nano Today.

[160] Du T, Zhang J, Li C, Song T, Li P, Liu J (2020). Gold/silver hybrid nanoparticles with enduring inhibition of coronavirus multiplication through multisite mechanisms. Bioconjug Chem.

[161] Pilaquinga F, Morey J, Torres M, Seqqat R, Piña MLN (2021). Silver nanoparticles as a potential treatment against SARS-CoV-2: a review. Wiley Interdiscip Rev Nanomed Nanobiotechnol.

[162] Jeremiah SS, Miyakawa K, Morita T, Yamaoka Y, Ryo A (2020). Potent antiviral effect of silver nanoparticles on SARS-CoV-2. Biochem Biophys Res Commun.

[163] He Q, Lu J, Liu N, Lu W, Li Y, Shang C, et al. Antiviral properties of silver nanoparticles against SARS-CoV-2: effects of surface coating and particle size. Nanomaterials (Basel). 2022;12(6). 10.3390/nano12060990 PMC8950764 35335803

[164] Alonso JCC, Delafiori J, Mariano VM, Dos Santos LA, Busanello ENB, Rocha AR (2021). Nano-immunotherapy accelerates recovery of patient with Covid-19: clinical analysis and metabolomics. J Phys Conf Ser.

[165] Youshia J, Ali ME, Stein V, Lamprecht A (2020). Nanoparticles' properties modify cell type-dependent distribution in immune cells. Nanomedicine.

[166] Huang Y, Lü X, Chen R, Chen Y (2020). Comparative study of the effects of gold and silver nanoparticles on the metabolism of human dermal fibroblasts. Regen Biomater.

[167] Fayek NM, Farag MA, Saber FR (2021). Metabolome classification via GC/MS and UHPLC/MS of olive fruit varieties grown in Egypt reveal pickling process impact on their composition. Food Chem.

[168] El-Newary SA, Afifi SM, Aly MS, Ahmed RF, El Gendy AEG, Abd-ElGawad AM, et al. Chemical profile of *Launaea nudicaulis* ethanolic extract and its antidiabetic effect in streptozotocin-induced rats. Molecules. 2021;26(4). 10.3390/molecules26041000 PMC7918448 33668635

[169] Ghini V, Maggi L, Mazzoni A, Spinicci M, Zammarchi L, Bartoloni A (2022). Serum NMR profiling reveals differential alterations in the lipoproteome induced by Pfizer-BioNTech vaccine in COVID-19 recovered subjects and naïve subjects. Front Mol Biosci.

[170] Dagla I, Iliou A, Benaki D, Gikas E, Mikros E, Bagratuni T, et al. Plasma metabolomic alterations induced by COVID-19 vaccination reveal putative biomarkers reflecting the immune response. Cells. 2022;11(7). 10.3390/cells11071241 PMC8997405 35406806

[171] Chen H, Ng JPM, Bishop DP, Milthorpe BK, Valenzuela SM (2018). Gold nanoparticles as cell regulators: beneficial effects of gold nanoparticles on the metabolic profile of mice with pre-existing obesity. J Nanobiotechnol.

[172] Kozics K, Sramkova M, Kopecka K, Begerova P, Manova A, Krivosikova Z, et al. Pharmacokinetics, biodistribution, and biosafety of PEGylated gold nanoparticles in vivo. Nanomaterials (Basel). 2021;11(7). 10.3390/nano11071702 PMC8305691 34203551

[173] Barreto A, Carvalho A, Campos A, Osório H, Pinto E, Almeida A (2020). Effects of gold nanoparticles in gilthead seabream—a proteomic approach. Aquat Toxicol.

[174] Alijagic A, Barbero F, Gaglio D, Napodano E, Benada O, Kofroňová O (2021). Gold nanoparticles coated with polyvinylpyrrolidone and sea urchin extracellular molecules induce transient immune activation. J Hazard Mater.

[175] Lee MJ, Lee SJ, Yun SJ, Jang JY, Kang H, Kim K (2016). Silver nanoparticles affect glucose metabolism in hepatoma cells through production of reactive oxygen species. Int J Nanomed.

[176] Rosário F, Duarte IF, Pinto RJB, Santos C, Hoet PHM, Oliveira H (2021). Biodistribution and pulmonary metabolic effects of silver nanoparticles in mice following acute intratracheal instillations. Environ Sci Pollut Res Int.

[177] Katarzyńska-Banasik D, Grzesiak M, Kowalik K, Sechman A (2021). Administration of silver nanoparticles affects ovarian steroidogenesis and may influence thyroid hormone metabolism in hens (*Gallus domesticus*). Ecotoxicol Environ Saf.

[178] Zhang W, Zhao Y, Li F, Li L, Feng Y, Min L (2018). Zinc oxide nanoparticle caused plasma metabolomic perturbations correlate with hepatic steatosis. Front Pharmacol.

[179] Siddiqui SA, Or Rashid MM, Uddin MG, Robel FN, Hossain MS, Haque MA, et al. Biological efficacy of zinc oxide nanoparticles against diabetes: a preliminary study conducted in mice. Biosci Rep. 2020;40(4). 10.1042/BSR20193972 PMC7138905 32207527

[180] Othman MS, Hafez MM, Abdel Moneim AE (2020). The potential role of zinc oxide nanoparticles in microRNAs dysregulation in STZ-induced type 2 diabetes in rats. Biol Trace Elem Res.

[181] Hassan RM, Elsayed M, Kholief TE, Hassanen NHM, Gafer JA, Attia YA (2021). Mitigating effect of single or combined administration of nanoparticles of zinc oxide, chromium oxide, and selenium on genotoxicity and metabolic insult in fructose/streptozotocin diabetic rat model. Environ Sci Pollut Res Int.

[182] Hu H, Guo Q, Fan X, Wei X, Yang D, Zhang B (2020). Molecular mechanisms underlying zinc oxide nanoparticle induced insulin resistance in mice. Nanotoxicology.

[183] El-Bahr SM, Shousha S, Albokhadaim I, Shehab A, Khattab W, Ahmed-Farid O (2020). Impact of dietary zinc oxide nanoparticles on selected serum biomarkers, lipid peroxidation and tissue gene expression of antioxidant enzymes and cytokines in Japanese quail. BMC Vet Res.

[184] Chen Z, Han S, Zheng P, Zhou D, Zhou S, Jia G (2020). Effect of oral exposure to titanium dioxide nanoparticles on lipid metabolism in Sprague-Dawley rats. Nanoscale.

[185] Zhang J, Shi J, Han S, Zheng P, Chen Z, Jia G (2022). Titanium dioxide nanoparticles induced reactive oxygen species (ROS) related changes of metabolomics signatures in human normal bronchial epithelial (BEAS-2B) cells. Toxicol Appl Pharmacol.

[186] Hu H, Li L, Guo Q, Zong H, Yan Y, Yin Y (2018). RNA sequencing analysis shows that titanium dioxide nanoparticles induce endoplasmic reticulum stress, which has a central role in mediating plasma glucose in mice. Nanotoxicology.

[187] Hu H, Zhang B, Li L, Guo Q, Yang D, Wei X (2020). The toxic effects of titanium dioxide nanoparticles on plasma glucose metabolism are more severe in developing mice than in adult mice. Environ Toxicol.

[188] Cai J, Zang X, Wu Z, Liu J, Wang D (2021). Altered protein S-glutathionylation depicts redox imbalance triggered by transition metal oxide nanoparticles in a breastfeeding system. NanoImpact.

[189] Mortensen NP, Pathmasiri W, Snyder RW, Caffaro MM, Watson SL, Patel PR (2022). Oral administration of TiO_2_ nanoparticles during early life impacts cardiac and neurobehavioral performance and metabolite profile in an age- and sex-related manner. Part Fibre Toxicol.

[190] He X, Jarrell ZR, Smith MR, Ly VT, Liang Y, Orr M (2023). Metabolomics of V_2_O_5_ nanoparticles and V_2_O_5_nanofibers in human airway epithelial BEAS-2B cells. Toxicol Appl Pharmacol.

[191] Cui L, Wang X, Zhao X, Sun B, Xia T, Hu S (2022). CeO2 nanoparticles induce pulmonary fibrosis via activating S1P pathway as revealed by metabolomics. Nano Today.

[192] Abulikemu A, Zhao X, Xu H, Li Y, Ma R, Yao Q (2023). Silica nanoparticles aggravated the metabolic associated fatty liver disease through disturbed amino acid and lipid metabolisms-mediated oxidative stress. Redox Biol.

[193] Al-Natour MA, Abdelrazig S, Ghaemmaghami AM, Alexander C, Kim DH (2022). Metabolic signatures of surface-modified poly(lactic-co-glycolic acid) nanoparticles in differentiated THP-1 cells derived with liquid chromatography-mass spectrometry-based metabolomics. ACS Omega.

[194] Nie P, Wang M, Zhao Y, Liu S, Chen L, Xu H. Protective effect of *Lactobacillus rhamnosus GG* on TiO_2_ nanoparticles-induced oxidative stress damage in the liver of young rats. Nanomaterials (Basel). 2021;11(3). 10.3390/nano11030803 PMC8004042 33801059

[195] Wang N, Tan HY, Li S, Xu Y, Guo W, Feng Y (2017). Supplementation of micronutrient selenium in metabolic diseases: its role as an antioxidant. Oxid Med Cell Longev.

[196] Kieliszek M, Lipinski B (2020). Selenium supplementation in the prevention of coronavirus infections (COVID-19). Med Hypotheses.

[197] Moghaddam A, Heller RA, Sun Q, Seelig J, Cherkezov A, Seibert L, et al. Selenium deficiency is associated with mortality risk from COVID-19. Nutrients. 2020;12(7). 10.3390/nu12072098 PMC7400921 32708526

[198] Aboulhoda BE, Abdeltawab DA, Rashed LA, Abd Alla MF, Yassa HD (2020). Hepatotoxic effect of oral zinc oxide nanoparticles and the ameliorating role of selenium in rats: a histological, immunohistochemical and molecular study. Tissue Cell.

[199] El-Demerdash FM, Nasr HM (2014). Antioxidant effect of selenium on lipid peroxidation, hyperlipidemia and biochemical parameters in rats exposed to diazinon. J Trace Elem Med Biol.

[200] Satyanarayana S, Sekhar JR, Kumar KE, Shannika LB, Rajanna B, Rajanna S (2006). Influence of selenium (antioxidant) on gliclazide induced hypoglycaemia/anti hyperglycaemia in normal/alloxan-induced diabetic rats. Mol Cell Biochem.

[201] van Bakel MM, Printzen G, Wermuth B, Wiesmann UN (2000). Antioxidant and thyroid hormone status in selenium-deficient phenylketonuric and hyperphenylalaninemic patients. Am J Clin Nutr.

[202] Zhang Q, Qian ZY, Zhou PH, Zhou XL, Zhang DL, He N (2018). Effects of oral selenium and magnesium co-supplementation on lipid metabolism, antioxidative status, histopathological lesions, and related gene expression in rats fed a high-fat diet. Lipids Health Dis.

[203] Oliveira LB, Mwangi VI, Sartim MA, Delafiori J, Sales GM, de Oliveira AN (2022). Metabolomic profiling of plasma reveals differential disease severity markers in COVID-19 patients. Front Microbiol.

[204] Li F, Fu L, Liu X, Liu X-a, Liang Y, Lv Y, et al. Serum metabolomic abnormalities in survivors of non-severe COVID-19. Heliyon. 2022;8(9):e10473. 10.1016/j.heliyon.2022.e10473 PMC9433334 36065322

[205] Meoni G, Ghini V, Maggi L, Vignoli A, Mazzoni A, Salvati L (2021). Metabolomic/lipidomic profiling of COVID-19 and individual response to tocilizumab. PLoS Pathog.

[206] Julkunen H, Cichońska A, Slagboom PE, Würtz P, Nightingale Health UK Biobank Initiative. Metabolic biomarker profiling for identification of susceptibility to severe pneumonia and COVID-19 in the general population. Elife. 2021;10:e63033. 10.7554/eLife.63033 PMC8172246 33942721

[207] Costanzo M, Caterino M, Fedele R, Cevenini A, Pontillo M, Barra L, et al. COVIDomics: the proteomic and metabolomic signatures of COVID-19. Int J Mol Sci. 2022;23(5). 10.3390/ijms23052414 PMC8910221 35269564

[208] Li Y, Hook JS, Ding Q, Xiao X, Chung SS, Mettlen M (2023). Neutrophil metabolomics in severe COVID-19 reveal GAPDH as a suppressor of neutrophil extracellular trap formation. Nat Commun.

[209] Bardanzellu F, Fanos V. Metabolomics, microbiomics, machine learning during the COVID-19 pandemic. Pediatr Allergy Immunol. 2022;33 (Suppl 27):86–8. 10.1111/pai.13640 PMC9303466 35080309

[210] Bennet S, Kaufmann M, Takami K, Sjaarda C, Douchant K, Moslinger E (2022). Small-molecule metabolome identifies potential therapeutic targets against COVID-19. Sci Rep.

[211] Kovarik JJ, Bileck A, Hagn G, Meier-Menches SM, Frey T, Kaempf A, et al. A multi-omics based anti-inflammatory immune signature characterizes long COVID-19 syndrome. iScience. 2023;26(1):105717. 10.1016/j.isci.2022.105717 PMC9719844 36507225

[212] Chen W, Yao M, Chen M, Ou Z, Yang Q, He Y (2022). Using an untargeted metabolomics approach to analyze serum metabolites in COVID-19 patients with nucleic acid turning negative. Front Pharmacol.

[213] Costa dos Santos Junior G, Pereira CM, Fidalgo TK, Valente AP. Saliva NMR-based metabolomics in the war against COVID-19. Anal Chem. 2020;92(24):15688–92. 10.1021/acs.analchem.0c04679 33215503

[214] Shoieb SM, El-Ghiaty MA, El-Kadi AOS (2021). Targeting arachidonic acid-related metabolites in COVID-19 patients: potential use of drug-loaded nanoparticles. Emergent Mater.

[215] Zheng H, Jin S, Li T, Ying W, Ying B, Chen D (2021). Metabolomics reveals sex-specific metabolic shifts and predicts the duration from positive to negative in non-severe COVID-19 patients during recovery process. Comput Struct Biotechnol J.

